# Multiscale topology in interactomic network: from transcriptome to antiaddiction drug repurposing

**DOI:** 10.1093/bib/bbae054

**Published:** 2024-03-14

**Authors:** Hongyan Du, Guo-Wei Wei, Tingjun Hou

**Affiliations:** College of Pharmaceutical Sciences, Zhejiang University, Hangzhou 310058, Zhejiang, China; Department of Mathematics, Michigan State University, MI 48824, USA; Department of Mathematics, Michigan State University, MI 48824, USA; Department of Electrical and Computer Engineering, Michigan State University, MI 48824, USA; Department of Biochemistry and Molecular Biology, Michigan State University, MI 48824, USA; College of Pharmaceutical Sciences, Zhejiang University, Hangzhou 310058, Zhejiang, China

**Keywords:** substance addiction, differentially expressed gene, persistent spectral theory, drug repurposing

## Abstract

The escalating drug addiction crisis in the United States underscores the urgent need for innovative therapeutic strategies. This study embarked on an innovative and rigorous strategy to unearth potential drug repurposing candidates for opioid and cocaine addiction treatment, bridging the gap between transcriptomic data analysis and drug discovery. We initiated our approach by conducting differential gene expression analysis on addiction-related transcriptomic data to identify key genes. We propose a novel topological differentiation to identify key genes from a protein–protein interaction network derived from DEGs. This method utilizes persistent Laplacians to accurately single out pivotal nodes within the network, conducting this analysis in a multiscale manner to ensure high reliability. Through rigorous literature validation, pathway analysis and data-availability scrutiny, we identified three pivotal molecular targets, mTOR, mGluR5 and NMDAR, for drug repurposing from DrugBank. We crafted machine learning models employing two natural language processing (NLP)-based embeddings and a traditional 2D fingerprint, which demonstrated robust predictive ability in gauging binding affinities of DrugBank compounds to selected targets. Furthermore, we elucidated the interactions of promising drugs with the targets and evaluated their drug-likeness. This study delineates a multi-faceted and comprehensive analytical framework, amalgamating bioinformatics, topological data analysis and machine learning, for drug repurposing in addiction treatment, setting the stage for subsequent experimental validation. The versatility of the methods we developed allows for applications across a range of diseases and transcriptomic datasets.

## INTRODUCTION

The ongoing drug addiction crisis presents a severe global public health challenge, particularly in the United States (US). The startling statistics from the US National Center for Drug Abuse Statistics shows that as of 2020, 37.309 million individuals aged 12 and older were identified as current illegal drug users. Among the diverse substances abused, opioids and cocaine are two predominant agents exacerbating the addiction epidemic.

The term ‘opioid’ encompasses both natural and synthetic substances that bind to specific opioid receptors within the human body. Misuse of opioids can lead to opioid use disorder (OUD), characterized by cravings, continued use despite physical and/or psychological decline, increased tolerance and withdrawal symptoms upon cessation [1]. While current medications effectively manage OUD to an extent, relapse and remission are prevalent due to the neurobiological shifts and opioid receptor tolerance from repeated abuse. The identification of new therapeutic targets and corresponding drug development is anticipated to alleviate the challenges encountered in OUD treatment. Cocaine is a tropane alkaloid stimulant known for its high addictive potential. Its misuse leads to serious health complications, including an increased risk of human immunodeficiency virus (HIV), hepatitis B and heart disease [2]. Despite ongoing research efforts, the FDA has not approved any effective medication for treating cocaine dependence.

Differentially expressed gene (DEG) analysis is a crucial tool in molecular biology and genetics, showing promise in identifying new targets for drug addiction treatment. Zhang *et al.* [3] have delineated biomarkers for opioid addiction through integrated bioinformatics analysis of gene expression data from heroin addicts. Similarly, Wang *et al.* [4] employed this approach to explore cocaine addiction and suggest potential therapeutic drugs targeting key genes, as identified through database analysis. Despite their valuable insights, these studies primarily rely on central algorithms for key gene identification from DEGs’ protein–protein interaction (PPI) networks. These algorithms focus predominantly on connection relationships within the network, often neglecting the quantitative assessment of interaction confidence or strength, thereby potentially missing critical information. Furthermore, these methods are traditionally limited in handling only the low-dimensional connections of data, which may result in an incomplete understanding of the complex, high-dimensional nature of PPI networks. Moreover, despite identifying key genes, these studies did not employ quantitative methodologies to drive drug discovery efforts targeting the identified genes.

Topological data analysis (TDA) introduces a groundbreaking perspective in analyzing these intricate biological networks. Within TDA, persistent homology (PH) stands out as a powerful tool, applying algebraic topology to uncover topological features such as holes and voids [5]. This technique facilitates a multiscale analysis through its filtration process, generating a series of topological invariants that uniquely characterize data. However, PH does not account for the homotopic shape evolution of data, a limitation addressed by persistent spectral graph (PST), also called persistent Laplacians [6]. PST encompasses both harmonic and non-harmonic spectra, where the former recovers all topological invariants from PH, and the latter reveals the homotopic shape evolution. It represents PPI networks in a topological space and adeptly captures both the overarching structure and subtle local interactions within the PPI network. PST has been successfully employed in various applications, including machine learning-assisted protein engineering predictions [7], forecasting dominant SARS-CoV-2 variants [8], predicting protein–ligand binding affinity [9], drug addiction analysis [10] and in dimensionality reduction for gene expression data [11].

**Figure 1:**
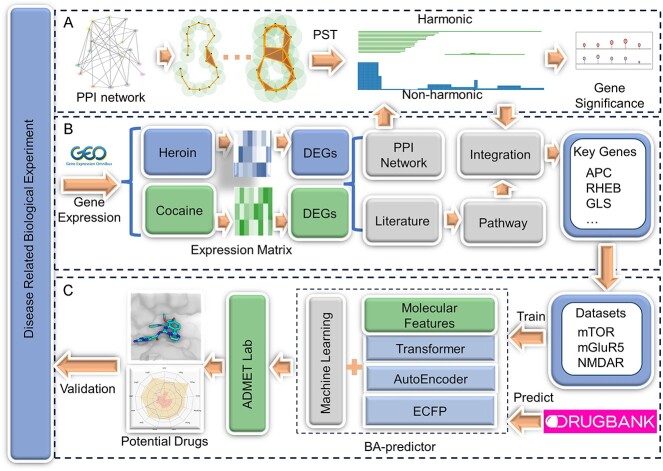
Overview of the Study: (**A**) PST-Based Topological Differentiation Analysis: This stage involves applying PST for topological differentiation analysis of the PPI network. The method quantifies the significance of nodes within the network in a multiscale manner. (**B**) DEG Analysis: We extracted opioid and cocaine addiction-related transcriptomic data from the GEO database. Key genes were identified from the PPI network derived from DEGs using topological analysis, and results were integrated across networks with various thresholds. Literature validation and pathway analysis were conducted to confirm the functionality and biological mechanisms of the key genes in substance addiction. (**C**) Drug Repurposing: Machine learning models, incorporating NLP-based fingerprints and traditional 2D fingerprints, were developed to predict the binding affinities of DrugBank compounds to three addiction-related targets: mTOR, mGluR5 and NMDAR. This process aims to identify potential repurposing candidates after the ADMET analysis for treating substance addiction.

In our study, we implemented a multifaceted strategy to bridge transcriptomic data analysis and drug discovery for substance addiction (Figure 1), employing PST for topological differentiation of PPI networks from DEG data. This approach effectively identifies key genes in opioid and cocaine addiction, offering multiscale analysis, quantitative interaction assessment and extraction of high-dimensional network information. After extensive validation, we identified mTOR, mGluR5 and NMDAR as significant targets for DrugBank repurposing. We developed machine learning models using NLP-based and traditional 2D fingerprints, demonstrating strong predictive accuracy in evaluating binding affinities of DrugBank compounds. This led to identifying drugs with effective binding energies and favorable ADMET profiles, making them promising candidates for further testing. Our comprehensive study not only sets a new standard in addiction treatment research but also offers a versatile framework applicable to various diseases, underscoring its potential in advancing drug discovery.

## RESULTS AND DISCUSSION

### Opioid addiction analysis

#### Differential expression analysis

**Figure 2:**
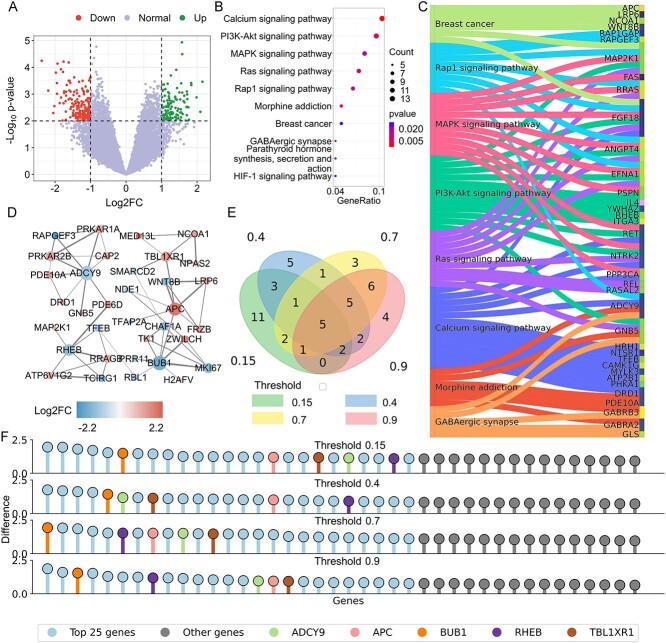
DEG analysis for opioid addiction. (**A**) Volcano plot of DEGs: This plot visually distinguishes DEGs in opioid addiction, highlighting significant genes above the threshold lines. (**B**) Enriched pathways in opioid addiction: Showcases the top 10 pathways significantly enriched in the context of opioid addiction, emphasizing their relevance in the disease mechanism. (**C**) Sankey plot of pathway-DEG relationships: Illustrates the top eight enriched pathways and their connections with DEGs, highlighting the intricate interplay between them. (**D**) Key gene-related PPI sub-network: Depicts the PPI sub-network specifically associated with key genes identified in opioid addiction. Each node represents a protein, and the size of the node is proportional to its degree, indicating the number of interactions it has within the network. (**E**) Venn diagram of significant intersections: Displays intersections of the top 25 significant genes across four PPI networks with varying thresholds, demonstrating the consistency of key genes in opioid addiction. (**F**) PST-based network differentiation significance: This graph presents the significance of individual genes as calculated from the PST-based differentiation of the network. For clarity, only the first 40 genes are included.

We obtained the GSE87823 dataset from the Gene Expression Omnibus (GEO) database to investigate the gene expression alterations associated with opioid addiction. This dataset encompasses expression data from the human nucleus accumbens, comparing heroin users with controls. We discerned a total of 295 DEGs in our study: 124 were up-regulated, while 171 were down-regulated (Figure 2A). This distinction provides us with an initial glimpse into the potential molecular mechanisms underlying opioid addiction, as genes that are differentially expressed can hint at pathways and processes that are altered in the disease state.

#### Multiscale topological differentiation of PPI networks

To further investigate the potential functional interactions among these DEGs, and to pinpoint those that may act as hubs or central players, we retrieved the PPI network for these DEGs from the STRING [12] database (Figure 2B). By establishing various interaction confidence thresholds (0.15, 0.4, 0.7 and 0.9), we facilitated a multi-resolution analysis. These thresholds enable us to identify robust interactions while also allowing for potential weaker, yet biologically relevant, interactions to be captured.

**Figure 3:**
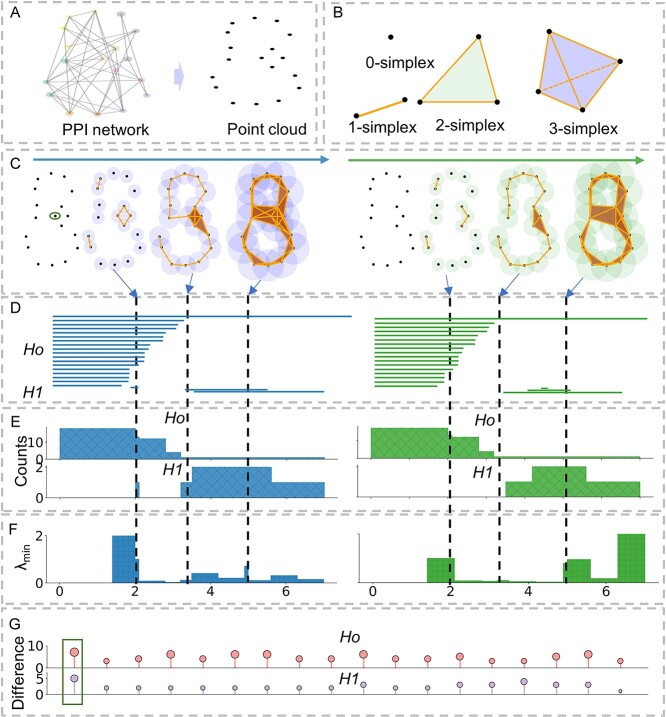
Topological differentiation of network. (**A**) PPI network as a point cloud: This panel visualizes the PPI network abstracted as a point cloud, forming the basis of a simplicial complex. (**B**) Basic unit of simplicial complex. (**C**) Filtration process: This panel depicts the filtration process, which generates a series of simplicial complexes with increasing radii. The left figure shows the original PPI network’s simplicial complex, while the right figure displays the new simplicial complex formed after deleting a protein (indicated by the green circle). PH and PST are used to characterize topological and geometric changes post-deletion. (**D**) Persistent barcodes in topological representation: PH is utilized here to provide a topological representation of the network, illustrated through persistent barcodes. The horizontal bars capture the persistence of topological features across the network’s filtration process. Specifically, the top series of bars represent 0-dimensional features (connected components), indicating how individual components merge over time, while the bottom series represent 1-dimensional features (loops or holes), illustrating the formation and closure of loops within the network. The left end of each bar marks the ’birth’ (appearance) of a feature at a specific scale, and the right end signifies its ’death’ (disappearance), with the length of the bar indicating the feature’s persistence across scales. (**E**) PST is applied to analyze the spectra of persistent Laplacians, with harmonic spectra indicating topological persistence, akin to PH. The figure shows changes in the count of topological invariants during filtration. (**F**) Capturing homotopic shape Evolution: The non-harmonic spectra in PST highlight the homotopic shape evolution of data. This panel demonstrates the change in the minimum of non-harmonic spectra during the filtration process. (**G**) Impact of node deletion on topological invariants: This figure illustrates the changes in topological invariants resulting from the deletion of each node in the network. The sum of changes during filtration is shown, with the green rectangle highlighting the changes corresponding to the most significant node.

At the core of our analytical framework is the novel method, we termed ‘multiscale topological differentiation of networks’. As depicted in Figure 3, this method can leverage persistent spectral theory and PH to provide a multidimensional analysis of the network’s topological and geometric characteristics. By constructing a simplicial complex through a carefully designed filtration process, our approach vividly captures the dynamic interplay of protein interactions across various scales. A key aspect of our methodology is the quantitative nature of the filtration process, based on the distance between proteins, derived from the confidence scores of each interaction pair. This precise and measurable analysis is a fundamental departure from conventional methods predominantly based on centrality algorithms, which often overlook such quantitative interaction information. By systematically removing specific proteins and observing the resulting topological and geometric shifts, we assess the network’s structural robustness and the critical role of individual proteins.

In this study, we employ PST to meticulously analyze the spectra of persistent Laplacians for each network. This analysis involves a detailed extraction of topological persistence from the harmonic spectra, complemented by insights into the homotopic shape evolution gleaned from the non-harmonic spectra. We then vectorize these extracted features, creating a comprehensive representation of the network’s topological characteristics. We calculate the Euclidean distance between vectorized features before and after the deletion of a protein. This distance serves as a metric for assessing the structural impact of the protein’s absence, thereby quantifying the protein’s importance within the network. Further enhancing our method’s precision, we rank the nodes in each network based on their importance under various threshold settings. By identifying and intersecting the top-ranked protein nodes across all four threshold-defined networks, we pinpoint key genes (Appendix A: Table S1, see Supplementary Data available online at http://bib.oxfordjournals.org/). This intersection approach ensures that the genes we classify as ‘key’ are consistently influential across multiple network scales, highlighting their potential critical role in the substance addiction-related biological processes under study. Applying this methodology, we have successfully identified six key genes that hold significant importance across the networks: APC, BUB1, ADCY9, RHEB and TBL1XR1 (Figure 2E and F). Their consistent presence across different network thresholds underscores their potential central role in addiction-related biological pathways.

To gain a more detailed understanding of the complex interactions between our identified key genes and opioid addiction, we undertook extensive literature validation and pathway enrichment analysis (Figure 2C and D). For a comprehensive overview of these findings, please see the Appendix B in supporting information (see Supplementary Data available online at http://bib.oxfordjournals.org/).

### Cocaine addiction analysis

**Figure 4:**
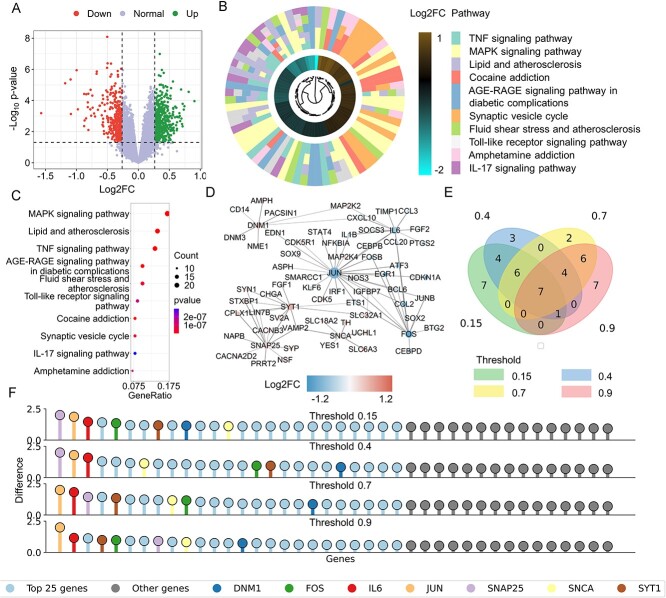
DEG analysis for cocaine addiction. (**A**) Volcano plot of DEGs: This plot visually distinguishes DEGs in cocaine addiction, highlighting significant genes above the threshold lines. (**B**, **C**) Enriched pathways in cocaine addiction: Showcases the top 10 pathways significantly enriched in the context of cocaine addiction, emphasizing their relevance in the disease mechanism. (**D**) Key gene-related PPI sub-network: Depicts the PPI sub-network specifically associated with key genes identified in cocaine addiction. Each node represents a protein, and the size of the node is proportional to its degree, indicating the number of interactions it has within the network. (**E**) Venn diagram of significant intersections: Displays intersections of the top 25 significant genes across four PPI networks with varying thresholds, demonstrating the consistency of key genes in cocaine addiction. (**F**) PST-based network differentiation significance: This graph presents the significance of individual genes as calculated from the PST-based differentiation of the network. For clarity, only the first 40 genes are included.

We procured and analyzed the GSE54839 dataset from the GEO database to scrutinize the alterations in gene expression consequential to cocaine addiction [13]. This dataset incorporates expression data derived from the human midbrain of chronic cocaine users ($n = 10$) juxtaposed against well-matched drug-free controls ($n = 10$). The midbrain, particularly the dopamine (DA)-synthesizing neurons therein, stands central to our study due to its pivotal role in addiction pathways. These neurons are largely implicated in mediating reward and pleasure centers in the brain, thereby making it crucial to discern the molecular perturbations induced by drug use, specifically in this neural locale. We discerned a total of 824 DEGs. Breaking down this data further revealed 467 up-regulated genes, indicating potential hyperactivity or over-expression in certain pathways, while 356 genes were observed to be down-regulated, signifying possible suppression or reduced activity (Figure 4A). To decipher the potential interplay between these DEGs, we consulted the STRING database to draw their PPI network (Figure 4D). Borrowing from our methodology deployed in the opioid addiction analysis, we leveraged PST to do topological differentiation analysis to identify key genes (Appendix A: Table S2, see Supplementary Data available online at http://bib.oxfordjournals.org/). These tools, with their distinct mechanisms, enabled us to spotlight pivotal genes within the network. Our differentiation analysis, which spanned multiple confidence thresholds, converged on seven key genes: DNM1, FOS, IL6, JUN, SNAP25, SNCA and SYT1 (Figure 4E and F).

To delve deeper into the intricate relationship between the key genes identified and cocaine addiction, we conducted thorough literature validation and pathway enrichment analysis (Figure 4D and C). Detailed findings and discussions on these aspects can be found in the supporting information (Appendix B, see Supplementary Data available online at http://bib.oxfordjournals.org/).

### Integrated analysis of ppioid and cocaine addiction DEGs

**Figure 5:**
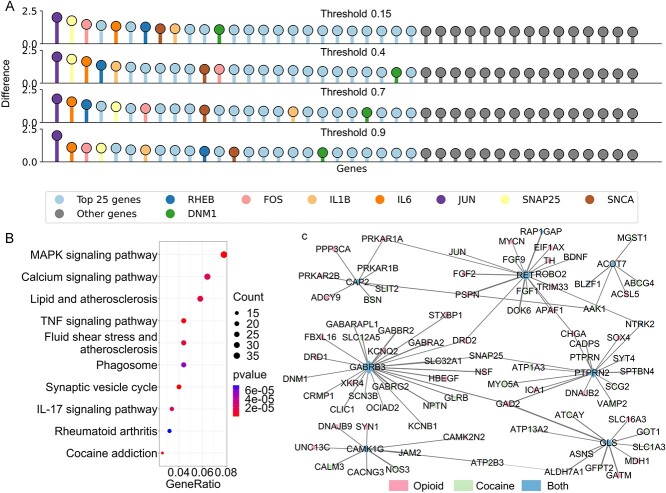
Integrated analysis of opioid and cocaine addiction DEGs. (**A**) PST-based network differentiation significance: This graph displays the significance of individual genes as determined by the PST-based differentiation in the integrated network of opioid and cocaine addiction DEGs. To enhance clarity, only the first 40 genes are shown. (**B**) Enriched pathways in integrated DEGs: Illustrates the pathways that are significantly enriched in the context of the integrated DEGs from both opioid and cocaine addiction, highlighting their combined biological relevance. (**C**) PPI sub-network related to common DEGs: Depicts the PPI sub-network associated with DEGs that are present in both opioid and cocaine addiction conditions, emphasizing the shared molecular mechanism. Each node represents a protein, and the size of the node is proportional to its degree, indicating the number of interactions it has within the network.

In our study to uncover common molecular mechanisms in opioid and cocaine addiction, we integrated DEGs from both conditions. This led to the identification of eight key genes shared across both addiction types: RHEB, DNM1, FOS, IL1B, IL6, JUN, SNAP25 and SNCA (Figure 5A). Notably, IL1B is a newly associated gene with these addictions, while RHEB had been previously linked to opioid addiction. The remaining six genes were identified in cocaine addiction analysis. Pathway enrichment on these integrated genes highlighted the MAPK signaling pathway as a critical factor in addiction (Figure 4B).

A targeted analysis on consistently differentially expressed genes in both addictions revealed ten genes: CAMK1G, GABRB3, ACOT7, CAP2, PTPRN2, RET, GLS, NTRK2, BLZF1 and RAP1GAP, with seven showing parallel expression changes in both conditions. This points to a potential shared molecular pathway influencing the pathophysiology of opioid and cocaine addictions (Figure 5C). Extensive literature validation reveals a significant association between GABRB3, PTPRN2, GLS and IL1B genes and drug addiction (Appendix B, see Supplementary Data available online at http://bib.oxfordjournals.org/).

### Repurposing of DrugBank for addiction-related targets

#### Binding affinity predictors for addiction-related targets

To find potential therapeutic agents for drug addiction, we utilized machine learning to repurpose drugs from DrugBank, targeting downstream proteins of key genes identified earlier. From our investigation, three proteins with substantial inhibitor data were identified: mTOR, NMDAR and mGluR5 [14–17]. We analyzed 4392 mTOR, 1777 mGlu5 and 2342 NMDAR inhibitors from the ChEMBL database, characterized by $\text{IC}_{50}$ or $K_{i}$ data (Appendix F, see Supplementary Data available online at http://bib.oxfordjournals.org/).

Employing transformer-based models, sequence-to-sequence autoencoders and traditional Extended-Connectivity Fingerprint (ECFP), we captured detailed molecular structures for machine learning analysis. These data were used to train Gradient Boosted Decision Trees (GBDT) models, predicting inhibitors’ activities against our target proteins. A consensus model approach was adopted to enhance prediction robustness, yielding Pearson correlation coefficients (R) of 0.876, 0.754 and 0.799, and root mean square errors of 0.758, 0.882 and 0.991 for mTOR, mGlu5 and NMDAR, respectively (Appendix H, see Supplementary Data available online at http://bib.oxfordjournals.org/).

#### Potential inhibitors of addiction-related targets in DrugBank

To discover potential inhibitors targeting addiction-related proteins, we deployed our machine learning predictors to gauge the binding affinity of various small molecules housed in the DrugBank database. In our analysis, we specifically selected molecules exhibiting a binding affinity greater than $-9.54$ kcal/mol (equivalent to a $K_{i}$ value of 100 nM). Such a stringent criterion, widely recognized in drug discovery, ensures that we consider only those compounds with a significant potential for therapeutic action [18].

##### Approved drugs with predicted efficacy on mTOR

We embarked on an evaluation of approved drugs that demonstrated strong binding affinity toward mTOR, with our selected affinity thresholds set at $-11$, $-10$ and $-9.54$ kcal/mol. In accordance with these thresholds, we identified 4, 7 and 7 drugs, respectively, that matched our criteria (Table 1).

**Table 1:** Summary of the FDA-approved drugs that are potential potent inhibitors of mTOR with binding affinity (BA) <$-9.54$ kcal/mol

DrugID	Name	Predicted BA (kcal/mol)
DB00877	Sirolimus	−12.46
DB00864	Tacrolimus	−12.32
DB01590	Everolimus	−12.07
DB00337	Pimecrolimus	−11.39
DB12483	Copanlisib	−10.40
DB11943	Delafloxacin	−10.31
DB09272	Eluxadoline	−10.31
DB01764	Dalfopristin	−10.12
DB00705	Delavirdine	−10.07
DB00709	Lamivudine	−10.05
DB00615	Rifabutin	−10.04
DB00879	Emtricitabine	−9.92
DB00210	Adapalene	−9.91
DB12153	Citicoline	−9.87
DB12767	Gaxilose	−9.84
DB13274	Micronomicin	−9.72
DB06725	Lornoxicam	−9.61
DB08907	Canagliflozin	−9.59

Significantly, both Sirolimus (Rapamycin) and Everolimus are recognized mTOR inhibitors [19, 20]. Sirolimus, the first mTOR inhibitor, was FDA-approved in 1999 for preventing transplant rejection in kidney recipients. Everolimus, while functionally akin to Sirolimus in inhibiting mTOR, boasts a more advantageous pharmacokinetic profile, with increased bioavailability and a faster terminal half-life. Tacrolimus and Pimecrolimus, both calcineurin inhibitors such as Sirolimus, share a macrocyclic lactone structure.

Copanlisib is a phosphoinositide 3-kinase (PI3K) inhibitor, prescribed for recurrent follicular lymphoma in adults who have undergone at least two prior treatments [21]. It selectively targets the alpha and delta isoforms of PI3K in malignant B-cells. Delafloxacin, a fluoroquinolone antibiotic, is used for certain adult bacterial infections, including skin infections and some pneumonias [22, 23]. Eluxadoline treats irritable bowel syndrome with diarrhea (IBS-D) in adults [24], working as an agonist on mu and kappa opioid receptors and an antagonist on delta opioid receptors. This modulates bowel activity and alleviates IBS-D symptoms by stabilizing intestinal contractility and countering stress-induced acceleration in the upper GI tract.

**Figure 6:**
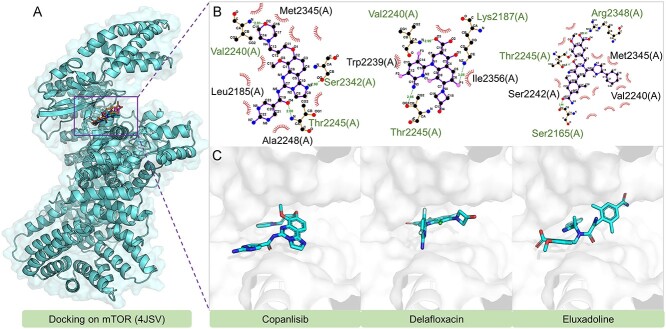
The docking structures and interactions of Copanlisib, Delafloxacin and Eluxadoline with mTOR.

To understand the inhibitory effects of Copanlisib, Delafloxacin and Eluxadoline on mTOR, we conducted molecular docking analysis, results of which are shown in Figure 6. These drugs demonstrate distinctive yet overlapping binding patterns to mTOR, particularly interacting with Thr2245. Copanlisib and Eluxadoline form hydrogen bonds with Thr2245 using their oxygen atoms, while Delafloxacin does so with a nitrogen atom. Additionally, Copanlisib and Delafloxacin also bond with Val2240 via oxygen atoms. Unique interactions include Copanlisib’s additional bond with Ser2342’s hydroxyl group, Delafloxacin’s connection with Lys2187 via a fluorine atom and Eluxadoline’s hydrogen bonds with both Arg2348 and Ser2165 through its oxygen atoms.

##### Investigational drugs with predicted efficacy on mTOR

In our investigation of drugs for mTOR efficacy, a higher proportion of investigational drugs showed greater affinity compared with approved ones, as detailed in Table 2, focusing on compounds with a binding affinity above $-11$ kcal/mol. Among these, Ridaforolimus specifically targets mTOR [25], while others are dual inhibitors of PI3K and mTOR. Notably, Omipalisib (GSK2126458 or GSK458) is under study for various cancers, including AML, showing notable anti-leukemia effects [26]. Gedatolisib (PF-05212384 or PKI-587) has progressed to clinical trials for different cancers and received FDA fast track designation for specific breast cancer treatments [27, 28]. Derived from Gedatolisib, PKI-179 is a dual PI3K and mTOR inhibitor, primarily developed for advanced malignant solid tumors [29].

**Table 2:** Summary of investigational drugs that have the potential to inhibit mTOR

DrugID	Name	Predicted BA (kcal/mol)
DB12703	Omipalisib	−13.06
DB11896	Gedatolisib	−12.36
DB13109	PKI-179	−12.34
DB11836	Sapanisertib	−12.20
DB12774	AZD-8055	−11.74
DB11925	Vistusertib	−11.50
DB13072	GDC-0349	−11.24
DB06233	Ridaforolimus	−11.21
DB12570	CC-223	−11.01

##### Approved drugs with predicted efficacy on mGluR5

In our analysis of DrugBank, we found that fewer approved drugs exhibited a high binding affinity for mGluR5 as compared with mTOR. Specifically, only two drugs, Doravirine and Desogestrel, displayed a binding affinity better than $-10$ kcal/mol, as detailed in Table 3.

**Table 3:** Summary of the FDA-approved drugs that are potential potent inhibitors of mGluR5 with BA <−9.54 kcal/mol

DrugID	Name	Predicted BA (kcal/mol)
DB12301	Doravirine	−10.52
DB00304	Desogestrel	−10.05
DB12612	Ozanimod	−9.90
DB01595	Nitrazepam	−9.87
DB06636	Isavuconazonium	−9.85
DB09291	Rolapitant	−9.78
DB08439	Parecoxib	−9.75
DB00294	Etonogestrel	−9.73
DB00475	Chlordiazepoxide	−9.72
DB11633	Isavuconazole	−9.68
DB00404	Alprazolam	−9.65
DB00904	Ondansetron	−9.63
DB15685	Selpercatinib	−9.62
DB11374	Amprolium	−9.57

Doravirine, an antiviral agent for HIV, acts as a non-nucleoside reverse transcriptase inhibitor (NNRTI) targeting HIV-1’s reverse transcriptase, crucial for viral replication [30]. Desogestrel, a synthetic progestin used in contraception, primarily inhibits ovulation [31]. Ozanimod, marketed as Zeposia, treats relapsing forms of multiple sclerosis [32] and ulcerative colitis [33], functioning by modulating the sphingosine-1-phosphate (S1P) receptor to reduce lymphocyte proliferation.

Molecular docking analysis was performed to investigate the binding mechanisms of Doravirine, Desogestrel and Ozanimod with the mGluR5 receptor, as depicted in Figure 8. A key finding is their hydrogen bonding interaction with Thr175: Doravirine uses its oxygen atom, while Desogestrel and Ozanimod use nitrogen atoms. This indicates Thr175’s potential importance in mGluR5 drug-target affinity. Additional analysis revealed Doravirine’s bond with Asp195 and Ozanimod’s notable interactions with Ser151, Ser152 and Gln198, while Desogestrel primarily exhibits hydrophobic interactions within the mGluR5 active site.

**Figure 7:**
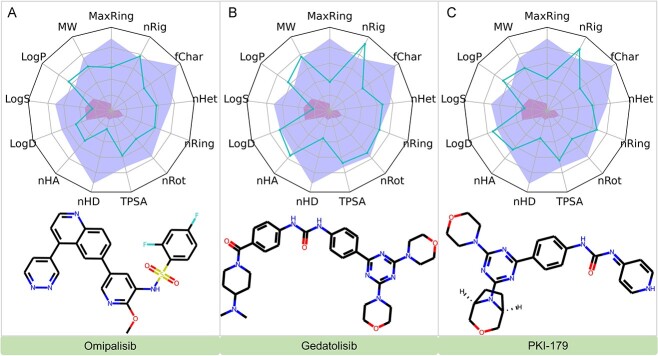
Evaluations of ADMET Properties for Omipalisib, Gedatolisib and PKI-179: this figure illustrates the ADMET profiles of Omipalisib, Gedatolisib and PKI-179, with the blue curves representing the values of 13 specified ADMET properties. The blue and red zones demarcate the upper and lower limits, respectively, of the optimal ranges for these properties.

##### Investigational drugs with predicted efficacy on mGluR5

In our subsequent analysis, we explored further investigational drugs projected to influence mGluR5 activity. It was observed that a greater number of these investigational drugs exhibited a higher affinity toward mGluR5 compared with approved drugs. Table 4 restrictively lists compounds boasting a binding affinity stronger than −10kcal/mol.

**Table 4:** Summary of investigational drugs that have the potential to inhibit mGluR5

DrugID	Name	Predicted BA (kcal/mol)
DB13004	Mavoglurant	−10.88
DB12733	Dipraglurant	−10.82
DB11649	Lersivirine	−10.40
DB12999	MK-6186	−10.17
DB15406	GLPG-0974	−10.11
DB04885	Cilansetron	−10.11
DB14929	Elsulfavirine	−10.10
DB13035	AG-24322	−10.02
DB12931	Fenobam	−10.01

Mavoglurant and Dipraglurant are notable as mGluR5 antagonists. Initially, Mavoglurant was explored for treating Fragile X syndrome [34] and levodopa-induced dyskinesia (LID) in Parkinson’s disease (PD) [35]. However, its clinical advancement was halted due to unmet primary endpoints in advanced trials. Conversely, Dipraglurant is designed to alleviate LID in PD patients [36]. Lersivirine (UK-453061), a pyrazole-based compound and part of the next-generation NNRTIs, was developed by ViiV Healthcare primarily to target HIV-1 infection [37].

##### Approved drugs with predicted efficacy on NMDAR

In our analysis of DrugBank, we identified only 7 approved drugs that exhibit a binding affinity greater than −9.54 kcal/mol to NMDAR (Table 5).

**Table 5:** Summary of the FDA-approved drugs that are potential potent inhibitors of NMDAR with BA <−9.54 kcal/mol

DrugID	Name	Predicted BA (kcal/mol)
DB00157	NADH	−10.10
DB03147	FAD	−10.09
DB00705	Delavirdine	−9.75
DB00131	Adenosine phosphate	−9.69
DB00118	Ademetionine	−9.61
DB00364	Sucralfate	−9.56
DB08874	Fidaxomicin	−9.56

Nicotinamide Adenine Dinucleotide (NADH) and Flavin Adenine Dinucleotide (FAD) are key coenzymes involved in various biochemical reactions. NADH shows potential benefits for conditions such as PD and chronic fatigue syndrome [38]. Delavirdine, sold as Rescriptor, is an NNRTI targeting HIV-1 [39], inhibiting the reverse transcriptase enzyme’s activities. Adenosine phosphate, initially a vasodilator and anti-inflammatory agent, now sees use in nutritional supplementation [40]. Ademetionine (SAMe) is a physiological methyl donor and a dietary supplement in the USA, known for mood and emotional well-being support [41].

Our docking studies with Delavirdine, Adenosine phosphate and Ademetionine at the NMDAR active site (Figure 10) revealed primary hydrophobic interactions. All three drugs form hydrogen bonds with Thr648, while Adenosine phosphate uniquely bonds with Thr647’s hydroxyl group.

**Figure 8:**
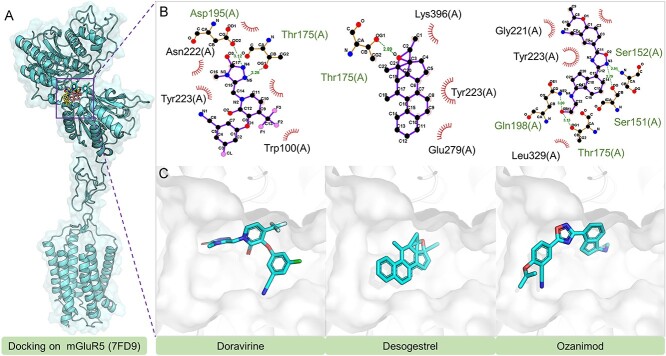
The docking structures and interactions of Doravirine, Desogestrel and Ozanimod with mGluR5.

##### Investigational drugs with predicted efficacy on NMDAR

In our pursuit to identify potential modulators of NMDAR, we explored further the realm of investigational compounds. Notably, a larger proportion of these compounds exhibited higher affinity for NMDAR in comparison to approved drugs. For brevity, only the top 10 of these compounds are presented in Table 6.

**Table 6:** Summary of top-10 investigational drugs that have the potential to inhibit NMDAR

DrugID	Name	Predicted BA (kcal/mol)
DB06741	Gavestinel	−11.70
DB12365	Perzinfotel	−10.84
DB12749	Butylphthalide	−10.47
DB05553	Regrelor	−10.23
DB12140	Dilmapimod	−10.14
DB03708	Adenosine 5’-phosphosulfate	−10.12
DB13019	Henatinib	−9.92
DB06334	Tucidinostat	−9.84
DB12012	PF-04457845	−9.83
DB05973	Inosine 5’-sulfate	−9.79

Gavestinel and Perzinfotel stand out as significant NMDAR antagonists among the investigated compounds. Gavestinel, intended for acute intracerebral hemorrhage management, acts as a non-competitive antagonist targeting the NMDA receptor’s glycine binding site. Notably, it exhibits over a 1000-fold selectivity for NMDAR compared with other receptors such as AMPA and kainate, and demonstrates oral bioavailability with *in vivo* activity [42]. Perzinfotel has been explored clinically for stroke treatment [43]. Butylphthalide, isolated from celery oil, shows promising therapeutic potential in hypertension and neuroprotection, especially in acute ischemic stroke [44, 45].

#### ADMET analysis

**Figure 9:**
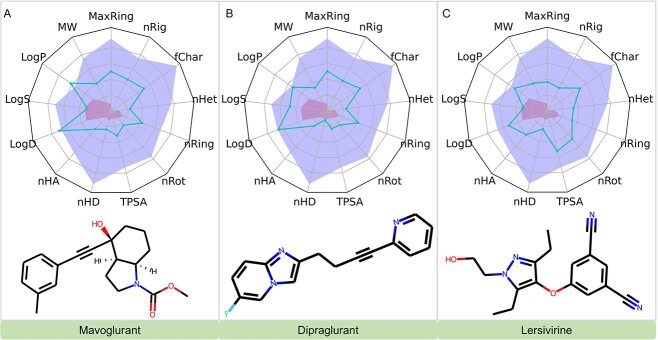
Evaluations of ADMET Properties for Mavoglurant, Dipraglurant and Lersivirine: this figure showcases the ADMET profiles for Mavoglurant, Dipraglurant and Lersivirine. The blue curves in the graph indicate the values for 13 specific ADMET properties of these compounds. The yellow and red zones in the graph are designated to highlight the upper and lower limits of the optimal ranges for each of these ADMET properties, respectively.

Our ADMET analysis [46], critical for the clinical viability of investigational drugs targeting mTOR, mGluR5 and NMDAR, revealed significant insights. Key highlights include Omipalisib’s favorable profile despite a high log *P*-value; Gedatolisib and PI3K facing solubility challenges (Figure 7); Mavoglurant exceeding solubility thresholds but maintaining acceptable ADMET properties; Dipraglurant and Lersivirine showing promising drug-like characteristics (Figure 9); and Gavestinel, Perzinfotel and Butylphthalide all displaying favorable ADMET profiles with specific notes on solubility and bioavailability (Figure 11). These findings are pivotal for the drugs’ further development in treating substance addiction. For detailed ADMET properties and comprehensive analysis, refer to the supporting information provided (Appendix C, see Supplementary Data available online at http://bib.oxfordjournals.org/).

**Figure 10:**
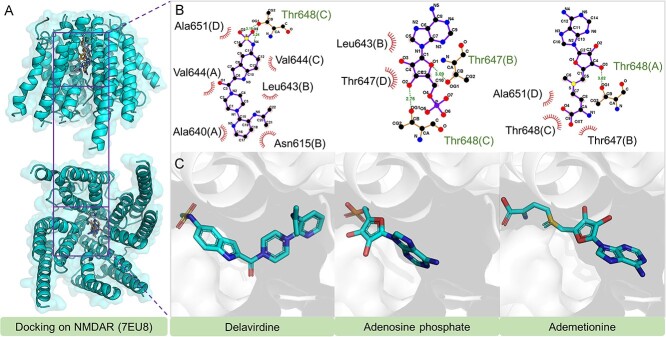
The docking structures and interactions of Delavirdine, Adenosine phosphate and Ademetionine with NMDAR.

**Figure 11:**
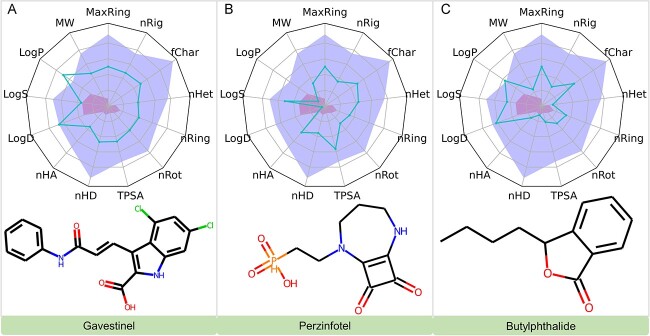
Evaluations of ADMET Properties for Gavestinel, Perzinfotel and Butylphthalide.

### Prospective insights into unvalidated targets for addiction therapy

In our detailed analysis targeting cocaine and opioid addiction, we have identified a number of intriguing targets such as BUB1, DNM1, JUN and SNAP25, which currently lack experimental validation in the context of addiction studies. These targets, surfaced through DEG analysis and topological network differentiation, present a unique opportunity to uncover potentially groundbreaking insights into the mechanisms of addiction and innovative treatment strategies. The potential of these unvalidated targets is significant, suggesting new pathways for drug action or revealing unexplored aspects of addiction biology. However, the road to harnessing their full potential is marked by the need for meticulous validation. This involves a combination of bioinformatics tools to predict interactions and functions, coupled with rigorous experimental studies to confirm their relevance to addiction and potential therapeutic impact. The complexity of addiction disorders, with their multifaceted biological underpinnings, requires a comprehensive and nuanced approach to the validation and application of these targets. Future research should foster a collaborative and interdisciplinary approach, uniting computational predictions with experimental biology to build a robust understanding of these targets. By navigating these challenges thoughtfully and systematically, and by encouraging the broader scientific community to embrace and support the exploration of these novel targets, we can potentially unlock new, effective strategies for understanding and treating substance addiction. The pursuit of these unexplored targets, therefore, represents not just a scientific challenge but also a promising frontier with the potential to significantly advance addiction research and therapy.

## METHODS

### DEG analysis and PPI network

We sourced gene expression datasets for DEG analysis from the GEO database, using Limma [47] for statistical identification of DEGs. For opioid addiction, dataset GSE87823 was analyzed, including only male samples aged 19–25, with quantile normalization and thresholds of |Log2FoldChange| $> 2$ and *P*-value $< 0.01$. For cocaine addiction, dataset GSE54839 [13] was used, incorporating all samples with pre-applied quantile normalization, setting |Log2FoldChange| $> 0.2630344$ and *P*-value $< 0.05$ as thresholds. Duplicates were removed, retaining only the highest expression probes, and probes linked to multiple genes were excluded. The PPI network was obtained from the STRING database [12], using four interaction confidence thresholds (0.15, 0.4, 0.7 and 0.9) for multi-resolution analysis.

### Multiscale topological differentiation of network

#### Persistent spectral theory and PH

We conceptualize the PPI network as a graph with nodes representing proteins and edges indicating interactions. Employing a simplicial complex allows for high-order interactions, capturing a broader range of shapes and high-dimensional relationships (Appendix D.1, D.2, see Supplementary Data available online at http://bib.oxfordjournals.org/). PST reveals multiscale structures by constructing a filtration—a nested sequence of simplicial complexes indexed by a parameter representing scale or threshold


(1) \begin{align*}& \emptyset = K_{0} \subseteq K_{1} \subseteq K_{2} \subseteq \cdots \subseteq K_{t} = K.\end{align*}

Here, $K$ is the largest simplicial complex achievable from the filtration, with each $K_{t}$ being a full simplicial complex at the filtration level $t$.

Accompanying this filtration is a corresponding sequence of chain complexes and boundary operators at each scale $t$


(2) \begin{align*}\cdots C_{q+1}^{t} \underset{\partial_{q+1}^{t^{\ast}}}{\overset{\partial_{q+1}^{t}}{\rightleftharpoons}} C_{q}^{t} \underset{\partial_{q}^{t^{\ast}}}{\overset{\partial_{q}^{t}}{\rightleftharpoons}} \cdots \underset{\partial_{3}^{t^{\ast}}}{\overset{\partial_{3}^{t}}{\rightleftharpoons}} C_{2}^{t} \underset{\partial_{2}^{t^{\ast}}}{\overset{\partial_{2}^{t}}{\rightleftharpoons}} C_{1}^{t} \underset{\partial_{1}^{t^{\ast}}}{\overset{\partial_{1}^{t}}{\rightleftharpoons}} C_{0}^{t} \underset{\partial_{0}^{t^{\ast}}}{\overset{\partial_{0}^{t}}{\rightleftharpoons}} \varnothing\end{align*}

where $C_{q}^{t}$ denotes the chain group for the sub-complex $K_{t}$ and $\partial _{q}^{t}$ is the $q$th boundary operator mapping from $C_{q}^{t}$ to $C_{q}^{t-1}$. For each $K_{t}$, every $q$-simplex is oriented, and the boundary operator $\partial _{q}^{t}$ is applied as follows:


\begin{align*} & \partial_{q}^{t}(\sigma_{q}) = \sum_{i}^{q}(-1)^{i}[v_{0}, \cdots, \hat{v_{i}},\cdots,v_{q}], \sigma_{q} \in K_{t}. \end{align*}

Here, $\sigma _{q} = [v_{0}, \cdots , v_{q}]$ is an oriented $q$-simplex within $K_{t}$, and $[v_{0}, \cdots , \hat{v_{i}},\cdots ,v_{q}]$ represents the oriented $(q-1)$-simplex obtained by omitting the $i$th vertex $v_{i}$ from $\sigma _{q}$.

Consider $\mathbb{C}_{q}^{t+p}$ to be the subset of $C_{q}^{t+p}$ consisting of chains whose boundaries are in $C_{q-1}^{t}$


(3) \begin{align*}& \mathbb{C}_{q}^{t+p} := \Big\{ \alpha \in C_{q}^{t+p} \ | \ \partial_{q}^{t+p}(\alpha) \in C_{q-1}^{t}\Big\}.\end{align*}

The $\eth _{q}^{t+p}$ is defined by


(4) \begin{align*}& \eth_{q}^{t+p}: \mathbb{C}_{q}^{t+p} \to C_{q-1}^{t}.\end{align*}

The $p$-persistent $q$-combinatorial Laplacian operator $\Delta _{q}^{t+p}$ along the filtration is given by


(5) \begin{align*}& \Delta_{q}^{t+p} = \eth_{q+1}^{t+p} \left(\eth_{q+1}^{t+p}\right)^{\ast} + \partial_{q}^{t^{\ast}} \partial_{q}^{t}.\end{align*}

The matrix representations of the boundary operators $\eth _{q+1}^{t+p}$ and $\partial _{q}^{t}$ are $\mathcal{B}_{q+1}^{t+p}$ and $\mathcal{B}_{q}^{t}$, respectively. The ${p}$-persistent ${q}$-combinatorial Laplacian matrix $\mathcal{L}_{q}^{t+p}$ is defined as


(6) \begin{align*}& \mathcal{L}_{q}^{t+p} = \mathcal{B}_{q+1}^{t+p} \Big(\mathcal{B}_{q+1}^{t+p}\Big)^{T} + \Big(\mathcal{B}_{q}^{t}\Big)^{T} \mathcal{B}_{q}^{t}.\end{align*}

This matrix is symmetric and positive semi-definite, ensuring that all eigenvalues are real and non-negative. The $p$-persistent $q$th Betti numbers, representing the number of $q$-cycles persisting in $K_{t}$ after $p$ filtration, correspond to the nullity of $\mathcal{L}_{q}^{t+p}$


(7) \begin{align*} \beta_{q}^{t+p} &= \text{dim}\Big(\mathcal{L}_{q}^{t+p}\Big) - \text{rank}\Big(\mathcal{L}_{q}^{t+p}\Big) = \text{nullity}\Big(\mathcal{L}_{q}^{t+p}\Big) \nonumber\\&= \boldsymbol{\#} \ \mathrm{of\ zero\ eigenvalues\ of }\ \mathcal{L}_{q}^{t+p}.\end{align*}

PST provides geometric insights from the spectra of this persistent combinatorial Laplacian, extending beyond mere topological persistence. Persistent Betti numbers offer information on topological constancy, while geometric transformations are discernible through the non-harmonic portions of the spectrum.

Complementing PST, PH offers a different approach to depict the persistence of topological invariants [5], though it primarily focuses on the harmonic spectral aspects of PST (Appendix D.3, see Supplementary Data available online at http://bib.oxfordjournals.org/).

#### Key gene identification via network topological differentiation

In our study, we employed PST and/or PH for network topological differentiation, assessing the significance of individual genes in a PPI network. Using the STRING database, we quantified interaction strengths to construct Rips complexes, facilitating a multi-resolution analysis of the network at various thresholds (0.15, 0.4, 0.7 and 0.9). We implemented ‘topological differentiation’ to observe changes in the network upon the removal of specific genes. This approach involves computing Euclidean distances between feature vectors of the original and perturbed networks, reflecting each gene’s impact on the network’s topological and geometric structure (Appendix D.4, see Supplementary Data available online at http://bib.oxfordjournals.org/).

### Machine learning-based drug repurposing

In our machine learning-based drug repurposing study, we prepared data from the ChEMBL database [48] and employed three fingerprint methodologies (bidirectional transformer-based model [49], sequence-to-sequence autoencoder [50] and 2D ECFPs [51]) to represent molecular structures. These fingerprints were used to build GBDT models for predicting binding affinities of inhibitors to mTOR, mGluR5 and NMDAR [52, 53]. A consensus approach, averaging predictions from all three models, was adopted for enhanced accuracy (Appendixes E, G, see Supplementary Data available online at http://bib.oxfordjournals.org/).

## CONCLUSION

Addressing the critical need for effective treatments in the escalating drug addiction crisis in the USA, our study presents a comprehensive strategy that transitions from transcriptomic data analysis to drug discovery. We aimed to identify potential drug candidates for opioid and cocaine addiction, aiming to alleviate the associated societal and health issues.

Our approach began with DEG analysis to identify key genes through innovative topological differentiation, ensuring reliability and multi-scale analysis. These targets underwent rigorous validation via pathway analysis and literature review, leading to the development of a machine learning-based drug repurposing study. We utilized Transformer and autoencoder embeddings alongside 2D fingerprints to screen drugs, focusing on their ADMET properties to assess therapeutic viability.

Three molecular targets—mTOR, mGluR5 and NMDAR—were identified as crucial in substance addiction. Our machine learning models effectively predicted binding affinities, identifying promising drugs from DrugBank with potential efficacy. These candidates, however, require further *in vivo* validation.

Our study advances computational methodologies in drug discovery, particularly in topological data analysis, and establishes a robust framework for future research. It highlights the translational potential of our methods, applicable to various diseases and transcriptomic data. Originally based on microarray analysis, our approach is also adaptable to scRNA-Seq data, addressing cellular heterogeneity critical in neurological diseases. This study sets new standards in addiction therapy and interdisciplinary drug discovery research, with the potential to transform healthcare research paradigms.

**Key Points**

- **Rigorous and Comprehensive Strategy:** Our work pioneers a rigorous approach that seamlessly integrates transcriptomic data analysis with drug discovery, addressing a critical need in addiction treatment research.
- **Topological Differentiation of PPI Network**: We have employed a novel method for the topological differentiation of the PPI network derived from DEGs.
- **Robust Machine Learning Models for Drug Repurposing**: Our study introduces robust machine learning models that significantly enhance the accuracy and efficiency of drug repurposing efforts.
- **Translational Potential**: Our methods provide a versatile framework applicable to diverse diseases and transcriptomic datasets, underscoring the potential to revolutionize drug discovery in various medical domains.

## Supplementary Material

supporting_information_re1_clean_bbae054

supporting_information_bbae054

## Data Availability

The data and source code of this study will be freely available at GitHub (https://github.com/Brian-hongyan/DEG-substance-addiction).

## SUPPORTING INFORMATION

Gene significance ranking resulted from topological differentiation (Appendix A); Literature validation and pathway enrichment (Appendix B); ADMET analysis for investigational drugs with therapeutic potential (Appendix C); Methods details for multiscale topological differentiation (Appendix D); Methods details for machine learning (Appendix E); Datasets utilized for machine learning and drug repurposing studies (Appendix F); Evaluation metric for machine learning model performance (Appendix G); Performance comparison of machine learning models (Appendix H). (PDF)

DEGs in opioid addiction (Sheet 1); DEGs in cocaine addiction (Sheet 2); Enriched pathways for opioid addiction and cocaine addiction, respectively (Sheet 3, 4); Datasets used for machine learning model training (Sheet 5, 6, 7); PPI networks derived from DEGs (Sheet 8, 9); ADMET properties for investigational drugs with therapeutic potential (Sheet 10). (XLSX)

## Author Biographies

**Hongyan Du** is currently a PhD student in the College of Pharmaceutical Sciences at Zhejiang University under the supervision of Prof. Tingjun Hou. His research interests mainly lie in the area of computer-aided drug design.

**Guo-Wei Wei** is an MSU foundation professor at the Department of Mathematics, Department of Biochemistry and Molecular Biology and Department of Electrical and Computer Engineering in Michigan State University. He received his PhD from the University of British Columbia in 1996. His research interests include mathematical foundations of data science and biosciences, deep learning, drug discovery and computational geometry, topology and graph.

**Tingjun Hou** is currently a professor in the College of Pharmaceutical Sciences at Zhejiang University. His research interests can be found at the website of his group: http://cadd.zju.edu.cn.
