# Supplementary material for: Multiscale topology in interactomic network: from transcriptome to antiaddiction drug repurposing
Source: Brief Bioinform. 2024 Mar 14;25(2):bbae054. doi: 10.1093/bib/bbae054 (PMC10948341; doi:10.1093/bib/bbae054)
Supplement: supporting_information_re1_clean_bbae054 [file supporting_information_re1_clean_bbae054.pdf]

Supporting Information for Multiscale Topology in Interactomic Network: From Transcriptome to Antiaddiction Drug Repurposing

Hongyan Du[1,2] and Guo-Wei Wei[2,3,4]*and Tingjun Hou[1†]
[1] College of Pharmaceutical Sciences,
Zhejiang University, Hangzhou 310058, Zhejiang, China.
[2] Department of Mathematics,
Michigan State University, MI 48824, USA.
[3] Department of Electrical and Computer Engineering,
Michigan State University, MI 48824, USA.
[4] Department of Biochemistry and Molecular Biology,
Michigan State University, MI 48824, USA.

January 4, 2024

*Corresponding author. Email: weig@msu.edu
†Corresponding author. Email: tingjunhou@zju.edu.cn

Contents

Appendices **1**

Appendix A Gene significance ranking **1**

Appendix B Literature validation and pathway enrichment **2**
- B.1 Opioid Addiction Analysis . . . 2
  - B.1.1 Literature validation . . . 2
  - B.1.2 Pathway enrichment . . . 3
- B.2 Cocaine Addiction Analysis . . . 4
  - B.2.1 Literature validation . . . 4
  - B.2.2 Pathway enrichment . . . 5
- B.3 Integrated Analysis of Opioid and Cocaine Addiction . . . 5
  - B.3.1 Literature validation . . . 5

Appendix C ADMET analysis **6**

Appendix D Multiscale topological differentiation **6**
- D.1 Simplicial complex and chain complex . . . 6
- D.2 q-combinatorial Laplacian . . . 8
- D.3 Persistent homology . . . 9
- D.4 Key gene identification via network topological differentiation . . . 9

Appendix E Machine learning-based drug repurposing **10**
- E.1 Data preparation . . . 10
- E.2 Molecular fingerprints . . . 10
  - E.2.1 Bidirectional transformer . . . 10
  - E.2.2 Sequence-to-sequence auto-encoder . . . 11
  - E.2.3 Extended-connectivity fingerprints . . . 11
- E.3 Machine learning models . . . 11

Appendix F Datasets for machine learning **12**

Appendix G Evaluation metrics **13**

Appendix H Performance comparison of machine learning models **14**

Appendix A Gene significance ranking

We employed topological differentiation to assess the significance of each gene within the PPI network through a multiscale analysis approach. Utilizing networks set at four different thresholds allowed us to conduct a comprehensive multi-resolution analysis. The key genes were identified by intersecting the top 25 genes from each of these four threshold-defined networks, ensuring a robust and consistent selection of significant genes (Table S1, S2).

Table S1: Top 25 Key Genes in the Opioid Addiction-Related DEG PPI Network. The key genes were identified as the intersection of the top-ranking genes across four networks, each defined by different threshold levels.

Ranking	Threshold 0.15	Threshold 0.4	Threshold 0.7	Threshold 0.9
1	YWHAZ	PML	**BUB1**	PML
2	MAP2K1	YWHAZ	PML	STAT6
3	NTRK2	EIF5B	EIF5B	**BUB1**
4	PGK1	PECAM1	STAT6	EIF5B
5	UBE2N	**BUB1**	NCOA1	NCOA1
6	**BUB1**	**ADCY9**	**RHEB**	LRP6
7	TPM2	NTRK2	SRD5A1	TP63
8	TP63	**TBL1XR1**	**APC**	**RHEB**
9	IL4	MCM5	**ADCY9**	SRD5A1
10	PPP3CA	NCOA1	MCM5	SORT1
11	MYH11	RPL35A	YWHAZ	WNT8B
12	REL	MKI67	**TBL1XR1**	ARNT
13	SOX5	IL4	PRKAR1A	CDKN1C
14	PECAM1	SIL1	TCAP	HSD17B6
15	PIDD1	RAPGEF3	NPAS2	**ADCY9**
16	**APC**	**APC**	TP63	**APC**
17	WDFY3	DOHH	DDX28	**TBL1XR1**
18	HMGB1	HBEGF	SORT1	NPAS2
19	**TBL1XR1**	STAT6	CDKN1C	RBM25
20	RAB40B	NPAS2	HSD17B6	DOHH
21	**ADCY9**	**RHEB**	RBM25	EIF5A2
22	MKI67	PRKAR1A	UBE2N	LUC7L3
23	RET	EIF5A2	RET	IL4
24	**RHEB**	PGK1	ARNT	NTRK2
25	PRDX3	NOC4L	H2AFV	EIF1AX

Table S2: Top 25 Key Genes in the Cocaine Addiction-Related DEG PPI Network. The key genes were identified as the intersection of the top-ranking genes across four networks, each defined by different threshold levels.

Ranking	Threshold 0.15	Threshold 0.4	Threshold 0.7	Threshold 0.9
1	**SNAP25**	**SNAP25**	**JUN**	**JUN**
2	**JUN**	**JUN**	**IL6**	**IL6**
3	**IL6**	**IL6**	**SNAP25**	VAMP2
4	GRIN1	BDNF	VAMP2	**SYT1**
5	**FOS**	GRIN1	**SYT1**	**FOS**
6	YWHAH	SYP	FGF2	CD44
7	BDNF	**SNCA**	BDNF	FGF2
8	**SYT1**	IL1B	CD44	**SNAP25**
9	GAD2	CD44	**SNCA**	EGR1
10	**DNM1**	SYN1	**FOS**	**SNCA**
11	MAST1	SOX2	EGR1	IRF1
12	SYP	GAP43	CDK5	IL1B
13	**SNCA**	CALM3	PAK1	CACNG2
14	ENO2	FGF2	SLC32A1	**DNM1**
15	IL1B	**FOS**	VCAM1	CDK5
16	STMN2	**SYT1**	GRIN1	PAK1
17	SNCB	SLC32A1	GAD2	CCL2
18	NTRK2	VAMP2	STXBP1	JUNB
19	SYN1	GAD2	**DNM1**	SLC6A3
20	GAP43	EDN1	BAG3	BAG3
21	ATP2B2	**DNM1**	CACNG2	CXCL10
22	GABRG2	STXBP1	CALM3	STXBP1
23	CALM3	KCNQ2	DNAJB1	CDKN1A
24	KCNQ2	TH	SYP	HSPA1A
25	SLC32A1	SNCB	HSPA1A	NCAN

Appendix B Literature validation and pathway enrichment

B.1 Opioid Addiction Analysis

B.1.1 Literature validation

To gain deeper insights into the intricate relationship between our identified key genes and opioid addiction, we turned to literature validation.

One of the intriguing genes that surfaced was RHEB, whose association with opioid addiction appears to be mediated through the mTORC1 pathway. RHEB, a small GTPase, is instrumental in activating mTORC1 [1]. While RHEB's binding to mTOR occurs distally from the kinase's active site, it induces a pronounced global conformational shift [2]. This allosteric modulation realigns active-site residues, enhancing catalytic actions. The significance of this interaction is underscored by the observed linkage between mTORC1 activation and opioid-induced phenomena such as tolerance and hyperalgesia. Diving deeper into the mechanisms, opioids have been postulated to induce alterations in protein translation within the nervous system [3]. These changes are believed to set the stage for the emergence of tolerance and hyperalgesia. Xu et al. unearthed that after repeated morphine administrations, mTOR—a regulator of protein translation—becomes activated in rat spinal dorsal horn neurons [3]. Notably, this mTOR activation is initiated through the μ opioid receptor and is channeled via the intracellular PI3K/Akt pathway. This discovery presents

mTOR inhibitors as potential therapeutic agents in stymieing or diminishing opioid tolerance, particularly when addressing chronic pain. Furthermore, the overarching role of mTORC1 extends beyond opioids. Other substances, including cocaine [4, 5], cannabinoids [6], and alcohol [7–9], have also been shown to activate mTORC1. This broad-spectrum influence underscores the pivotal role mTORC1 assumes in the realm of substance addiction.

ADCY9 is an integral protein implicated in the morphine addiction pathway, playing a pivotal role in the cellular signaling cascade. Its primary function is to catalyze the conversion of ATP into cyclic AMP (cAMP) [10]. One of the most noteworthy roles of cAMP is its ability to activate protein kinase A (PKA). When cAMP binds to the regulatory subunits of PKA, it triggers a conformational change, releasing the catalytic subunits. Upon release, these catalytic subunits are rendered active and embark on phosphorylating a myriad of protein targets inside the cell, initiating or modulating various cellular processes. A noteworthy aspect of PKA's function lies in its capability to modulate the dynamics of gamma-aminobutyric acid (GABA) [11–14]. PKA not only influences the secretion of GABA by neuronal cells [12, 13] but also plays a role in fine-tuning the functional responsiveness of GABA receptors [11, 14]. GABA stands out as the principal inhibitory neurotransmitter within the central nervous system [15, 16]. It is instrumental in modulating neuronal excitability, thus maintaining a delicate balance between excitatory and inhibitory signals in the brain. A malfunction or dysregulation in ADCY9's activity could unleash a cascade of molecular events that may disturb this delicate balance. Such an aberration could potentially compromise GABAergic signaling, culminating in a state of hyperexcitability within the central nervous system. This heightened excitability is often accompanied by a surge in the release of dopamine, the neurotransmitter associated with pleasure and reward [17, 18].

The adenomatous polyposis coli (APC) protein is intrinsically tied to opioid addiction through its interactions within the Wnt signaling pathway. Research has highlighted the Wnt pathway's involvement in withdrawal symptoms ensuing from opioid receptor activation, either due to exposure to morphine or as a result of chronic inflammation [19]. Moreover, the manifestation of opioid-induced hyperalgesia, a paradoxical increase in pain sensitivity, has been tied to the activities of reactive astrocytes, which are intriguingly governed by Wnt5a signaling [20]. APC plays a pivotal role in this milieu by acting as a negative regulator of the Wnt pathway. It is a key constituent of the destruction complex, a molecular assembly that also includes the proteins AXIN and Glycogen Synthase Kinase 3β (GSK-3β) [21, 22]. Within the destruction complex, GSK-3β phosphorylates specific serine and threonine residues on β-catenin which marks β-catenin for ubiquitination and subsequent degradation.

TBL1XR1 is implicated in opioid addiction through its potential involvement in the Wnt signaling pathway. Some mutations can amplify the activation of the Wnt pathway mediated by TBL1XR1 [23]. While there are indications of its role, a deeper exploration is essential to fully demonstrate TBL1XR1's significance in the context of opioid addiction.

B.1.2 Pathway enrichment

To gain a deeper insight into the broader biological processes influenced by the DEGs and to pinpoint significant biological pathways, we conducted pathway enrichment analysis. The three most prominent pathways are the Calcium signaling, PI3K-Akt pathway, and MAPK pathway, involving 13, 12, and 10 DEGs, respectively. Notably, six DEGs have been identified in Morphine addiction pathway.

The intricate link between opioid addiction and calcium signaling has been extensively researched. Upon ligand binding to the opioid receptor, there is a resultant dissociation of the α-GTP complex from the γ dimer subunits. This γ dimer subsequently acts to directly inhibit the calcium channels, leading to a reduction in the intracellular calcium concentration [24]. The modulatory effect of opioid receptor activation on calcium channel activity has been corroborated across various brain regions, including the hippocampus, nucleus locus coeruleus, and the area postrema, among others [25]. König et al. found that PI3Kγ

modulates the desensitization of the mu opioid receptor [26]. Madishetti et al. discovered the essential function of PI3Kγ in cAMP-mediated inflammatory hypernociception [27]. The Mitogen-Activated Protein Kinase (MAPK) pathway is closely associated with opioid addiction. Several genes within MAPK pathway have been identified in relation to opioid receptor signaling and behavior, including ERK 1/2 [28], c-Jun N-terminal kinase [29] and p38 MAPK [30].

B.2 Cocaine Addiction Analysis

B.2.1 Literature validation

To unravel the intricate interplay between our pinpointed key genes and cocaine addiction, we conducted a detailed literature validation for a comprehensive understanding.

The immediate early gene FOS (c-Fos) has been a significant player in the context of cocaine addiction, as underscored by numerous studies exploring its intricate involvement in cocaine-induced neuroplasticity and behavioral responses [31–35]. Zhang et al. investigated the influence of repeated cocaine administration on the expression of various molecular markers, particularly in Fos-deficient brain environments [32]. The result showed that Fos's absence in the brain results in altered expression levels of several transcription factors, neurotransmitter receptors, and intracellular signaling molecules —all of which are induced by repeated cocaine exposure. This observation suggests that Fos is instrumental in acquiring cocaine-induced persistent changes. Xu demonstrated that the dendritic reorganization of medium spiny neurons, a hallmark of cocaine-induced neuroadaptations, was notably attenuated in Fos-mutant brains [35]. These structural changes, or the lack thereof, were not merely restricted to the molecular domain; they manifested behaviorally as well. Mice with mutant FOS genes exhibited a marked reduction in behavioral sensitization, a phenomenon characterized by an escalating response to repeated cocaine administration.

Interleukin-6 (IL-6) has been suggested to have some connections with cocaine-induced behaviors. Mai et al.'s study on IL-6 knockout mice indicated a potential protective effect against cocaine-induced reactions, hinting at a role of the JAK2/STAT3 and PACAP signaling pathways [36]. Additionally, observed changes in serum levels of IL-6 among cocaine users hint at a peripheral response to the drug [37]. However, the overall evidence linking IL-6 to cocaine effects is still limited, and more research is needed to establish a direct relationship.

Research suggests that SYT1 may influence cognitive performance from cocaine addiction. Silva et al. investigated the link between SYT1-rs2251214 and susceptibility as well as severity (as gauged by the addiction severity index) of CUD in a study involving 315 smoked cocaine addicts and 769 non-addicts [38]. Their findings highlighted a significant association between SYT1-rs2251214 and CUD vulnerability. Additionally, Viola et al. identified a correlation between cognitive performance and SYT1-rs2251214 in women diagnosed with cocaine use disorder [39].

Research has increasingly highlighted the significant role of α-synuclein (SNCA) in various forms of substance addiction, extending beyond cocaine. SNCA is known for its critical involvement in dopaminergic transmission, a pathway often implicated in addictive behaviors [40]. Qin et al. observed that chronic cocaine abuse leads to an upregulation of SNCA expression in the human striatum [41]. This was evidenced by immunoblot analysis in the ventral putamen, revealing elevated SNCA protein levels in striatal synaptosomes of cocaine users compared to age-matched drug-free controls. Additionally, a study by Foroud et al. suggests a link between SNCA variations and alcohol craving [42]. Although alcohol craving is a common feature of alcohol dependence, it is not universally present. Their findings indicate that genetic variation in SNCA could contribute to these craving behaviors, underscoring the gene's broader relevance in substance addiction.

B.2.2 Pathway enrichment

In an effort to elucidate the underlying biological processes linked to cocaine addiction, we undertook a comprehensive pathway enrichment analysis. Notably, the Cocaine and Amphetamine addiction pathways emerge as direct correlates to substance dependency. At the forefront of these pathways are the MAPK signaling cascade, the Lipid metabolism and atherosclerosis pathway, and the TNF signaling axis. It is of particular significance that the MAPK signaling cascade, previously implicated in opioid addiction studies, reemerges here, accentuating its pivotal role in the broader context of substance addiction [43, 44].

The association of cocaine with the Lipid metabolism and atherosclerosis pathway underscores the drug's deleterious cardiovascular implications. Many studies have documented the profound vascular influence of cocaine intake, emphasizing inflammation and atherosclerosis as predominant systemic outcomes with both acute and chronic manifestations [45, 46]. Not much literature evidence of relations between TNF signaling pathway and cocaine addiction was found. The only exception was due to Lewitus et al. who demonstrated that microglial TNF-α can modulate cocaine-induced neural plasticity and behavioral sensitization [47].

B.3 Integrated Analysis of Opioid and Cocaine Addiction

B.3.1 Literature validation

Extensive literature validation underscores a significant association between GABRB3, PTPRN2, and GLS genes with drug addiction. The GABRB3 gene encodes the β 3 subunit of the GABAA receptor. Chen et al. highlighted the potential of GABRB3 in heroin dependence, suggesting that its elevated expression may play a pivotal role in the disorder's pathogenesis [48]. Furthermore, several studies have emphasized the role of GABRB3 in alcoholism. Noble et al. demonstrated that both DRD2 and GABRB3 variants heightened the risk for alcoholism [49]. Similarly, findings by Song et al. linked paternal transmission of GABRB3 to alcoholism [50], while Young and colleagues associated both DRD2 and GABRB3 with alcohol-related expectations [51]. PTPRN2 has emerged as a significant gene in substance dependence and cognitive behavior [52]. Linkage analyses have identified its significance in the comorbidity of cocaine dependence and major depressive episodes in humans [53]. Further, genome-wide association studies have linked PTPRN2 to cognitive performance, risk-taking behaviors, and smoking initiation [54]. Complementing the human findings, studies on PTPRN2 knockout mice have reported reduced concentrations of crucial neurotransmitters such as dopamine, norepinephrine, and serotonin in the brain [55]. Glutaminase (GLS) is an enzyme responsible for the conversion of the amino acid glutamine into glutamate [56]. As the predominant excitatory neurotransmitter in the central nervous system, glutamate plays pivotal roles in the behavioral effects elicited by psychostimulant drugs [57]. Over the past two decades, a combination of fundamental neuroscience research and preclinical studies using animal models has underscored the centrality of glutamate transmission in drug reward mechanisms, reinforcement, and the propensity for relapse [58]. Given that glutaminases are the primary producers of glutamate in the brain, their potential significance in the realm of drug addiction becomes apparent [57]. Glutamate's effects are mediated through two primary receptor types: ionotropic and metabotropic [59]. The former are ion channels that facilitate ion movement upon glutamate activation, whereas the latter, being G-protein coupled receptors, trigger intricate signal transduction pathways when bound to glutamate. Notably, among these, NMDAR (an ionotropic receptor) and mGluR5 (a metabotropic receptor) are crucial in modulating neuronal excitability. Multiple studies have illuminated their intimate association with substance addiction [60–66]. As for IL1B, acute cocaine exposure has been linked to increased IL1B levels in specific brain regions, as shown by Cearley et al.'s findings in the cortex and nucleus accumbens [67]. Montesinos et al. further observed that cocaine-induced changes in CX3CL1 concentrations are related to IL1B levels, suggesting activation of shared inflammatory pathways in the hippocampus [68]. The significance of IL-1B in addiction is further underscored by the findings of Liang et al., who explored its genetic aspects in relation to alcohol dependence [69]. Their study

revealed that polymorphisms in IL-1B are associated with a modified risk profile for alcohol dependence. Intriguingly, the particular single nucleotide polymorphisms (SNPs) at positions -511 and -31 are found with greater frequency in opioid-dependent populations. Although the association is described as weak, it nonetheless suggests a potential genetic predisposition linked to an increased risk of opioid dependence. This aligns with our identification of IL1B as a key gene in addiction, revealing its cross-substance implications and reinforcing its potential role as a biomarker or therapeutic target.

Appendix C ADMET analysis

Understanding that a molecule's pharmacokinetic and safety profiles are pivotal in its clinical trial success, we focused on the ADMET (absorption, distribution, metabolism, excretion, and toxicity) properties of our identified investigational drugs. A comprehensive understanding of these properties is crucial, as drug candidates can fail in advanced stages if they do not meet ADMET criteria, which govern their behavior within the human body [70]. Utilizing the ADMETlab 2.0 tool, we assessed 13 distinct ADMET attributes for each drug which show potential efficacy on the three targets [70].

For mTOR inhibitors, our analysis revealed that Omipalisib has a logP value slightly exceeding the optimal range, which can potentially affect its solubility and permeability. However, its other ADMET properties fell within acceptable limits, suggesting a favorable pharmacokinetic profile overall. On the other hand, Gedatolisib presented some challenges, with a relatively large molecular weight and a higher number of rings and hydrogen bond donors. These structural features contribute to its high logP value, resulting in poor water solubility. Similarly, while PI3K, developed from Gedatolisib, has a smaller molecular weight, it inherits the challenge of a high logP value and limited water solubility.

In the case of mGluR5 inhibitors, we found that Mavoglurant's logP and logD values surpassed recommended thresholds, indicating potential issues with water solubility. However, its other ADMET properties were within acceptable ranges. Dipraglurant and Lersivirine, on the other hand, showed well-balanced ADMET profiles, suggesting favorable drug-like characteristics for these compounds.

For potential NMDAR therapeutic agents, Gavestinel, Perzinfotel, and Butylphthalide, were also systematically predicted. The results revealed that all three compounds exhibited favorable ADMET properties. Gavestinel, with its moderate molecular weight, showed a slightly elevated logP value attributed to its hydrophobic groups, leading to marginal water solubility limitations. Despite this, Gavestinel retained its oral bioavailability. Contrarily, Perzinfotel exhibited enhanced water solubility due to the presence of a phosphate group within its molecular structure, which concomitantly resulted in reduced logP and logD values. Lastly, Butylphthalide presented with a logP value on the verge of the upper limit, yet all other properties fell within the scientifically recommended and acceptable range, demonstrating its optimal characteristics.

In conclusion, this comprehensive ADMET analysis offers crucial insights into the pharmacokinetic and safety profiles of these investigational drugs. These findings are instrumental in guiding their further development and optimization for potential use in treating substance addiction.

Appendix D Multiscale topological differentiation

D.1 Simplicial complex and chain complex

The PPI network is conceptualized as a graph where nodes correspond to proteins, and edges denote the interactions between pairs of proteins. Extending beyond the graph structure, we employ the concept of a simplicial complex to allow high-order interactions in the network, encapsulating a more comprehensive range of shapes and facilitating the inclusion of high-dimensional topological relationships. A simplicial

complex is an aggregate of simplices, which are the fundamental building blocks that span various dimensions. Specifically, a q-simplex (denoted as σ_q) can be defined as the convex combination of $(q+1)$ vertices $(v_0, v_1, v_2, ..., v_q)$ that are affinely independent, expressed as:

$$\sigma_q := [v_0, v_1, ..., v_q]. \tag{1}$$

In the realm of Euclidean geometry, simplices correspond to familiar shapes: a 0-simplex represents a point, a 1-simplex forms a line segment, a 2-simplex constitutes a triangle, and a 3-simplex is akin to a tetrahedron. For any given set of $(q+1)$ points, every non-empty subset can be the vertices of a subsimplex, considered to be a face of a q-simplex, denoted as $\sigma^m \subset \sigma_q$. The structure known as a simplicial complex, denoted by K, is defined as a finite set of simplices that adhere to two critical conditions:

1) Any face of a simplex within K must also be included in K;
2) The nonempty intersection of any two simplices is a face of both simplices.

The relationship between simplices within a complex can be characterized by their adjacency, a concept extended from the combinatorial graph theory. In a graph, the degree of a vertex, denoted as deg(v), is the tally of its adjacent edges. This principle becomes more complex when applied to q-simplices, which are adjacent to both $(q-1)$-simplices and $(q+1)$-simplices. To navigate this complexity, it is necessary to distinguish between upper and lower adjacencies for defining the degree of a q-simplex when $q > 0$. Two q-simplices, σ_q^1 and σ_q^2, within a complex K are considered upper adjacent $\sigma_q^1 \overset{U}{\sim} \sigma_q^2$ if they both constitute faces of the same $(q+1)$-simplex. Conversely, they are lower adjacent $\sigma_q^1 \overset{L}{\sim} \sigma_q^2$ if they share a common $(q-1)$-simplex. The upper degree, deg$_U(\sigma_q)$, is the count of $(q+1)$-simplices of which the q-simplex is a face, while the lower degree, deg$_L(\sigma_q)$, is the count of its $(q-1)$-simplex faces, which invariably equals $(q+1)$. Consequently, for q-simplices where $q > 0$, the degree is the sum of the upper and lower degrees:

$$\text{deg}(\sigma_q) = \text{deg}_L(\sigma_q) + \text{deg}_U(\sigma_q). \tag{2}$$

The concept of orientation in a simplex is pivotal and is dictated by the sequence of its vertices, excluding the 0-simplex which has no orientation. For a q-simplex σ_q, two given vertex orderings are considered to be similarly oriented if they can be interchanged by an even permutation, hence both configurations represent an orientation of σ_q. If an odd permutation is required, the orderings are dissimilarly oriented. Consequently, an oriented$q$-simplex is the pairing of a simplex σ_q with a designated orientation. When every simplex within a simplicial complex K is assigned an orientation, we refer to K as an oriented simplicial complex.

In topological, geometric, and algebraic studies, the framework of chain complexes is essential. Consider a simplicial complex K with a maximum dimension of q. Within this context, a q-chain is constructed as a formal summation of the q-simplices in K, utilizing coefficients drawn from the $\mathbb{Z}_2$ field. The collection of all q-chains, under the addition defined by $\mathbb{Z}_2$, forms a group known as the chain group, symbolized by $C_q(K)$. To create connections between chain groups across various dimensions, the boundary operator for q-chains, denoted ∂_q, serves as a mapping function from $C_q(K)$ to $C_{q-1}(K)$. This operator transforms a q-chain, represented as a linear combination of q-simplices, into the corresponding combination of their $(q-1)$-dimensional boundaries. For a q-simplex spanned by vertices $[v_0, v_1, \cdots, v_q]$, symbolized as σ_q, the action of ∂_q is mathematically articulated as:

$$\partial_q \sigma_q = \sum_{i=0}^{q} (-1)^i [v_0, \cdots, \hat{v}_i, \cdots, v_q], \tag{3}$$

where $\hat{v}_i$ indicates the $(q-1)$-simplex formed by excluding the vertex v_i from σ_q. A q-chain that yields a zero boundary upon the application of ∂_q is termed a q-cycle.

The structure of a chain complex is characterized by a sequence of chain groups linked by boundary operators. This sequence forms a continuous cascade from higher-dimensional chains down to zero-dimensional chains, which is mathematically depicted as:

$$\cdots \xrightarrow{\partial_{q+2}} C_{q+1}(K) \xrightarrow{\partial_{q+1}} C_q(K) \xrightarrow{\partial_q} C_{q-1}(K) \xrightarrow{\partial_{q-1}} \cdots \tag{4}$$

In this expression, $C_q(K)$ represents the chain group composed of q-chains in the simplicial complex K, while ∂_q denotes the boundary operator mapping q-chains to $(q-1)$-chains. The chain complex terminates with the null set, indicating that the boundary of a 0-chain is inherently zero since there are no negative-dimensional chains. Each boundary operator connects adjacent levels of the complex, ensuring that the boundary of a boundary within this sequence is always zero, a foundational concept in homology theory.

D.2 q-combinatorial Laplacian

In the field of algebraic topology, the boundary operators within a simplicial complex K facilitate the construction of a bridge between different dimensions of chains. The matrix representation of the q-boundary operator $\partial_q : C_q(K) \longrightarrow C_{q-1}(K)$ is denoted as $\mathcal{B}_q$. The dimensions of this matrix—rows corresponding to the number of $(q-1)$-simplices and columns to the number of q-simplices—reflect the structure of K. The adjoint of ∂_q, symbolized as $\partial_q^* : C_{q-1}(K) \longrightarrow C_q(K)$, operates in the reverse direction of the boundary operator, and its matrix representation is the transpose of $\mathcal{B}_q$, labeled $\mathcal{B}_q^T$. Diving deeper into topological invariants, the q-combinatorial Laplacian is a linear transformation $\Delta_q : C_q(K) \longrightarrow C_q(K)$ defined as:

$$\Delta_q := \partial_{q+1}\partial_{q+1}^* + \partial_q^*\partial_q. \tag{5}$$

Its matrix form, $\mathcal{L}_q$, emerges as a summation of the product of boundary matrices and their transposes:

$$\mathcal{L}_q = \mathcal{B}_{q+1}\mathcal{B}_{q+1}^T + \mathcal{B}_q^T\mathcal{B}_q. \tag{6}$$

Specifically, for $q = 0$, the combinatorial Laplacian, also known as the graph Laplacian, simplifies due to ∂_0 being a zero map, resulting in:

$$\mathcal{L}_0 = \mathcal{B}_1\mathcal{B}_1^T. \tag{7}$$

The matrix elements of $\mathcal{L}_q$ are defined by the relationships between simplices and their degrees of connectivity:

$$(\mathcal{L}_q)_{ij} = \begin{cases} \deg(\sigma_q^i) + q + 1, & \text{if } i = j. \\ 1, & \text{if } i \neq j,\, \sigma_q^i \overset{U}{\nsim} \sigma_q^j \text{ and } \sigma_q^i \overset{L}{\sim} \sigma_q^j \text{ with similar orientation.} \\ -1, & \text{if } i \neq j,\, \sigma_q^i \overset{U}{\nsim} \sigma_q^j \text{ and } \sigma_q^i \overset{L}{\sim} \sigma_q^j \text{ with dissimilar orientation.} \\ 0, & \text{if } i \neq j \text{ and either },\, \sigma_q^i \overset{U}{\sim} \sigma_q^j \text{ or } \sigma_q^i \overset{L}{\nsim} \sigma_q^j. \end{cases} \tag{8}$$

For the graph Laplacian, when $q = 0$, the matrix $\mathcal{L}_0$ is simplified as:

$$(\mathcal{L}_0)_{ij} = \begin{cases} \deg(\sigma_0^i), & \text{if } i = j. \\ -1, & \text{if } \sigma_0^i \overset{U}{\sim} \sigma_0^j. \\ 0, & \text{otherwise.} \end{cases} \tag{9}$$

The q-combinatorial Laplacian matrix is a pivotal element in understanding the intrinsic topological features of a simplicial complex. Due to its symmetric and positive semi-definite nature, it is guaranteed that all eigenvalues of the matrix are real and non-negative. These spectral properties play a central role in revealing the topological invariants of the underlying space. According to the combinatorial Hodge theorem, the Betti numbers, which serve as topological invariants representing the number of q-dimensional holes in

a space, can be computed from the spectrum of the Laplacian. The multiplicity of the zero eigenvalue, also known as the nullity of the Laplacian matrix $\mathcal{L}_q$, corresponds to the q-th Betti number β_q:

$$\beta_q = \text{dim}(\mathcal{L}_q(K)) - \text{rank}(\mathcal{L}_q(K)) = \text{nullity}(\mathcal{L}_q(K)) = \#\text{ of zero eigenvalues of } \mathcal{L}_q(K). \tag{10}$$

These Betti numbers encapsulate crucial topological information. β_0 indicates the number of connected components within the complex. β_1 represents the number of one-dimensional or "circular" holes, often conceptualized as tunnels or loops. β_2 corresponds to the number of two-dimensional voids or "cavities", akin to the hollow spaces enclosed by surfaces.

D.3 Persistent homology

Complementing PST, PH offers a different approach for multiscale analysis through filtration techniques [71]. In this framework, for a given simplicial complex K, we define the q-cycle group $\mathcal{Z}_q$ and the q-boundary group $\mathcal{B}_q$ as the kernel and image of boundary operators ∂_q and ∂_{q+1}, respectively. The equations

$$\mathcal{Z}_q = \text{Ker}\partial_q = \{c \in C_q \mid \partial_q c = 0\}, \tag{11}$$

$$\mathcal{B}_q = \text{Im}\partial_{q+1} = \{c \in C_q | \exists d \in C_{q+1} : c = \partial_{q+1} d\}, \tag{12}$$

describe these groups, where C_q represents the set of q-chains. Since $\partial_q \circ \partial_{q+1} = 0$, it follows that $\mathcal{B}_q \subseteq \mathcal{Z}_q \subseteq C_q$. Consequently, we define the q-homology group $\mathcal{H}_q$ as the quotient group:

$$\mathcal{H}_q = \mathcal{Z}_q / \mathcal{B}_q. \tag{13}$$

The qth Betti number β_q is then the rank of $\mathcal{H}_q$. Furthermore, the persistence of these topological features is quantified by the qth persistence Betti number $\beta_q^{i,j}$, which is the rank of the homology groups of K_i that persist to K_j, formulated as

$$\beta_q^{i,j} = \text{rank}(\mathcal{Z}_q(K_i)/(\mathcal{B}_q(K_j) \cap \mathcal{Z}_q(K_i))). \tag{14}$$

Through filtration, PH provides a detailed perspective on the persistence of topological invariants, though it primarily focuses on the harmonic spectral aspects of PST.

D.4 Key gene identification via network topological differentiation

We can make use of PST and/or PH for topological differentiation analysis to assess the significance of individual genes within a PPI network. Using the STRING database, we quantify the interaction strength between protein pairs with a combined score. This score serves as a basis for formulating an abstract distance between proteins, which facilitates the construction of a Rips complex. To conduct a multi-resolution and multiscale analysis, we utilize four PPI network using thresholds of 0.15, 0.4, 0.7, and 0.9. The network can be denoted as $G = (V, E)$. Within this framework, we use a concept known as 'topological perturbation analysis', proposed by Chen et al. [72], at the m-th vertex v_m by considering the subgraph G_m that results from eliminating v_m and all edges linked to v_m. For a specified threshold T, we define the distance between two proteins v_i and v_j in G as:

$$D_{ij} = \begin{cases} 1 - s_{ij}, & \text{if } s_{ij} > T. \\ \infty, & \text{otherwise.} \end{cases} \tag{15}$$

where s_{ij} denote the combined score of interaction between proteins v_i and v_j in STRING database. In this study, PST is utilized to derive both topological and geometric characteristics of the network in its original and perturbed states. For the analysis, a Rips complex-based filtration up to two dimensions is established.

In each network case, ten filtration parameters are evenly chosen from the interval 0 to $(1 - T)$. From each Laplacian spectrum, we compute the harmonic spectra count and the five statistical descriptors (minimum, maximum, mean, standard deviation, and sum) of the non-harmonic spectra. For every network G, we encapsulate its attributes into a vector f_G, defined as $f_G = \Theta(G)$. Correspondingly, after perturbation at vertex v_m, we obtain the feature vector f_{G_m} for each G_m, defined as $f_{G_m} = \Theta(G_m)$, where Θ encapsulates PST analysis and network vectorization. We quantify the significance of the node v_m by computing the Euclidean distance between feature vectors f_G and f_{G_m}:

$$S_m^G = distance(f_G, f_{G_m}). \tag{16}$$

This metric reflects the impact of gene v_m within the PPI network G by indicating how its removal alters the network's structure in terms of topology and geometry.

Appendix E Machine learning-based drug repurposing

E.1 Data preparation

For our machine learning models, we sourced inhibitor datasets pertaining to mTOR, mGluR5 and NMDAR from the ChEMBL database [73]. These datasets comprise SMILES strings of molecular compounds, each paired with a bioactivity label. The initial labels assigned to these data points were either IC_{50} or K_i values. To adapt these experimental labels into binding affinities (BAs) suitable for our models, we employed the conversion formula: BA $= 1.3633 \times \log_{10} K_i$(kcal/mol). IC_{50} labels were subsequently estimated to K_i values based on the relationship $K_i = IC_{50}/2$, in alignment with recommendations by Kalliokoski [74]. For instances where a single molecule had multiple bioactivity value labels, we computed the average of these labels. In addition, we retrieved small molecule drugs, categorized under either approved or investigational status, from the DrugBank database (version 5.1.10) [75]. To ensure consistency, their SMILES strings were canonicalized by the RDKit toolkit.

E.2 Molecular fingerprints

In our molecular analysis, we employed three fingerprinting methodologies to delineate molecular structures. Two of them harness advanced NLP techniques: one utilizing a bidirectional transformer-based model [76] and the other deploying a sequence-to-sequence autoencoder framework [77]. These NLP-driven methodologies leverage pretrained models to transduce canonical SMILES notations into 512-dimensional latent vectors. Complementing these, our study also incorporated a classical topological approach characterized by the 2D ECFP [78], synthesized via the comprehensive RDKit computational toolkit.

E.2.1 Bidirectional transformer

Chen and colleagues innovatively crafted a self-supervised learning (SSL) framework for the purpose of pretraining deep neural networks using millions of unlabeled molecular structures [76]. This methodology yields latent vectors derived from input SMILES, encapsulating essential molecular structural details, thereby serving as robust fingerprints for subsequent machine learning applications. The core of their SSL platform employs the bidirectional encoder transformer (BET) model, which leverages attention mechanisms for enhanced accuracy. During the pretraining phase on the SSL platform, the process involved the formation of data pairs comprising authentic SMILES strings and their masked counterparts, with a specific fraction of symbols intentionally obscured. To create these pairs, 15% of the symbols in all SMILES strings were masked. In this masking process, 80% of the symbols were fully obscured, 10% were left unaltered, and the remaining 10% were randomly modified. The employment of the attention mechanism in the BET model ensures that the significance of each symbol within the SMILES string is fully captured. In

their study, Chen et al. utilized SMILES strings extracted from one or a combination of the ChEMBL [73], PubChem [79], and ZINC [80] databases for training their SSL-based BET model. In the context of the current study, we opted to utilize the fingerprints generated directly from the pretrained model on ChEMBL database, without any additional fine-tuning (termed as TF-FPs).

E.2.2 Sequence-to-sequence auto-encoder

A novel unsupervised learning approach, utilizing a sequence-to-sequence autoencoder, has been developed to interpret molecular information encapsulated within the SMILES representation [77]. By translating one molecular format into another, the model efficiently compresses detailed chemical structures into a latent space positioned between the encoder and decoder components. During this transformation, intermediary vectors embed significant physicochemical details, enabling the pretrained model to retrieve molecular descriptors from input SMILES strings without additional training. The encoder employs a combination of convolutional neural network (CNN) and recurrent neural network (RNN) structures, directing their outputs to form intermediary vector representations. On the other hand, the decoder, which is mainly based on RNN architectures, interprets these vectors to produce the desired output. To enhance the richness of the latent vectors, an auxiliary classification module is incorporated, forecasting specific molecular properties. The loss function, instrumental during the autoencoder model's training, is a fusion of cross-entropies associated with the decoder's probabilistic character outputs and mean squared errors pertinent to molecular property predictions. This comprehensive model has been rigorously trained on expansive datasets sourced from the ZINC and PubChem databases.

E.2.3 Extended-connectivity fingerprints

Extended-connectivity fingerprints (ECFPs) are a unique class of topological fingerprints used for molecular characterization, primarily developed for structure-activity modeling [78]. The ECFP approach assigns specific identifiers to each atom in a molecule based on their properties and surrounding atoms. Through iterative hashing techniques, these identifiers are updated to encompass the molecule's neighborhood information, and redundant features are removed. Ultimately, these identifiers are consolidated into a bit array, capturing the molecule's distinctive features. We employed the RDKit library to produce ECFPs. This library constructs circular fingerprints using the Morgan algorithm, which necessitates a radius parameter dictating the algorithm's iteration count. In our study, the ECFP radius was set to 2, with the fingerprint length designated at 2048.

E.3 Machine learning models

We adopted the GBDT algorithm for constructing our machine learning models [81, 82]. GBDT can be utilized for both regression and classification tasks. The algorithm operates by initially utilizing a weak learner, predominantly a decision tree, to offer primary predictions, subsequently quantifying the residual errors between these initial predictions and the actual outcomes. A subsequent decision tree is tailored to model these residuals, with the explicit objective of correcting inaccuracies introduced by the preceding trees in the sequence. This iterative methodology continues, with each iteration aiming to refine the residuals from the preceding step, until the total tree count attains a predetermined number or the cumulative residuals satisfy a specified threshold. The final model is an aggregate of all the individual trees, which are used for inference on new datasets.

In our investigation, we utilized three molecular fingerprint modalities, namely TF-FP, AE-FP, and ECFP, to represent inhibitor molecules. Three distinct machine learning models were subsequently curated using the GBDT framework. To augment the predictive accuracy and robustness of the models in assessing BAs for mTOR, mGluR5 and NMDAR, a consensus-based approach was adopted. This consensus model combined

the predictions by averaging the binding affinity predictions sourced from each of the three individual models.

Appendix F Datasets for machine learning

In our study, we developed predictive models for binding affinity using inhibitor datasets sourced from the ChEMBL database (Table S3). These models were then applied to estimate the binding affinity of various drugs listed in DrugBank towards three specific targets. The compounds within these datasets were represented using SMILES strings, and their corresponding binding affinities, measured in kcal/mol, were used as the target labels for model training. The distribution of these binding affinity labels across each dataset is depicted in Figure S1. In our analysis, we evaluated six key properties of the molecules within the training set. These properties encompass the molecular weight, LogP, and the count of heavy atoms. Additionally, we assessed the structural complexity by determining the number of rings, as well as the potential for molecular interaction and stability by counting the number of hydrogen bond donors and acceptors (Figure S2).

Table S3: The summary of datasets used in this study.

Dataset	Protein name	Dataset size	Binding affinity range (kcal/mol)
mTOR	Mammalian target of rapamycin	4392	[-5.86, -14.25]
mGluR5	The metabotropic glutamate receptor subtype 5	1777	[-5.47, -13.23]
NMDAR	N-methyl-D-aspartate receptor	2342	[-5.45, -13.32]

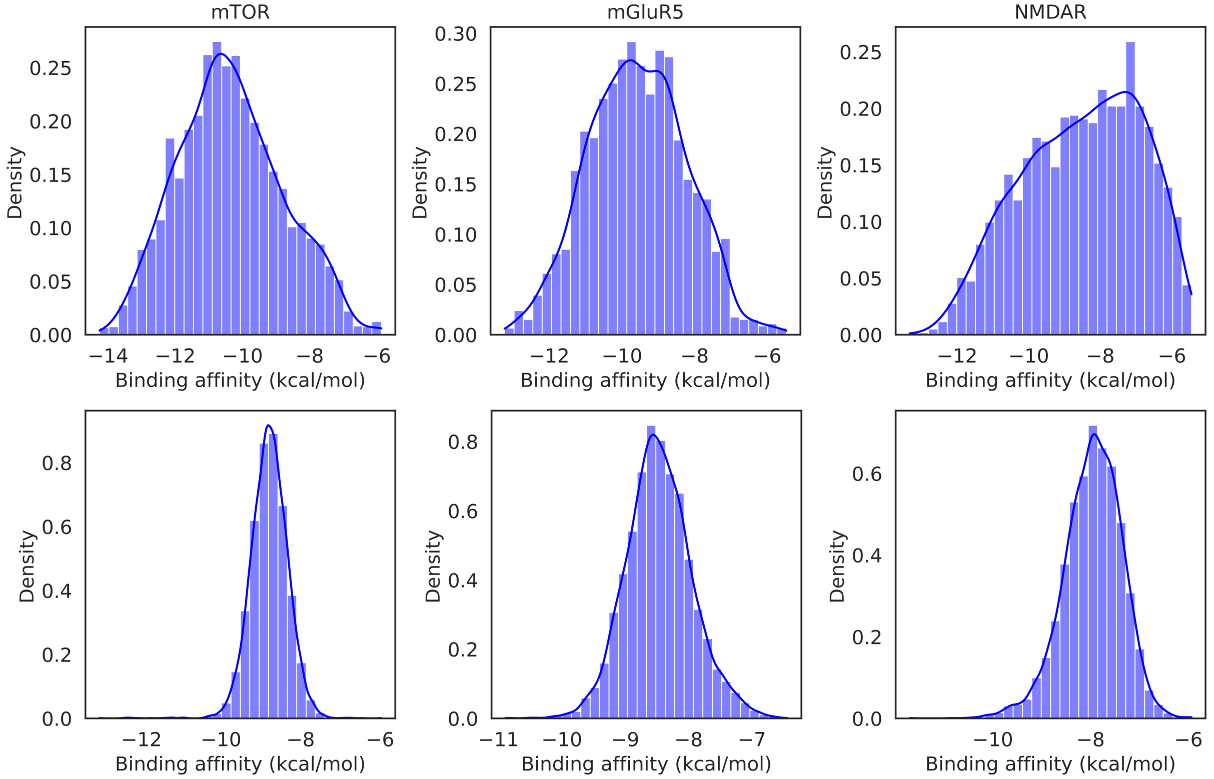

Figure S1: Binding Affinity Distributions in Datasets: The top panel displays the distribution of binding affinity values extracted from the ChEMBL dataset. The bottom panel illustrates the distribution of predicted binding affinities for drugs in DrugBank.

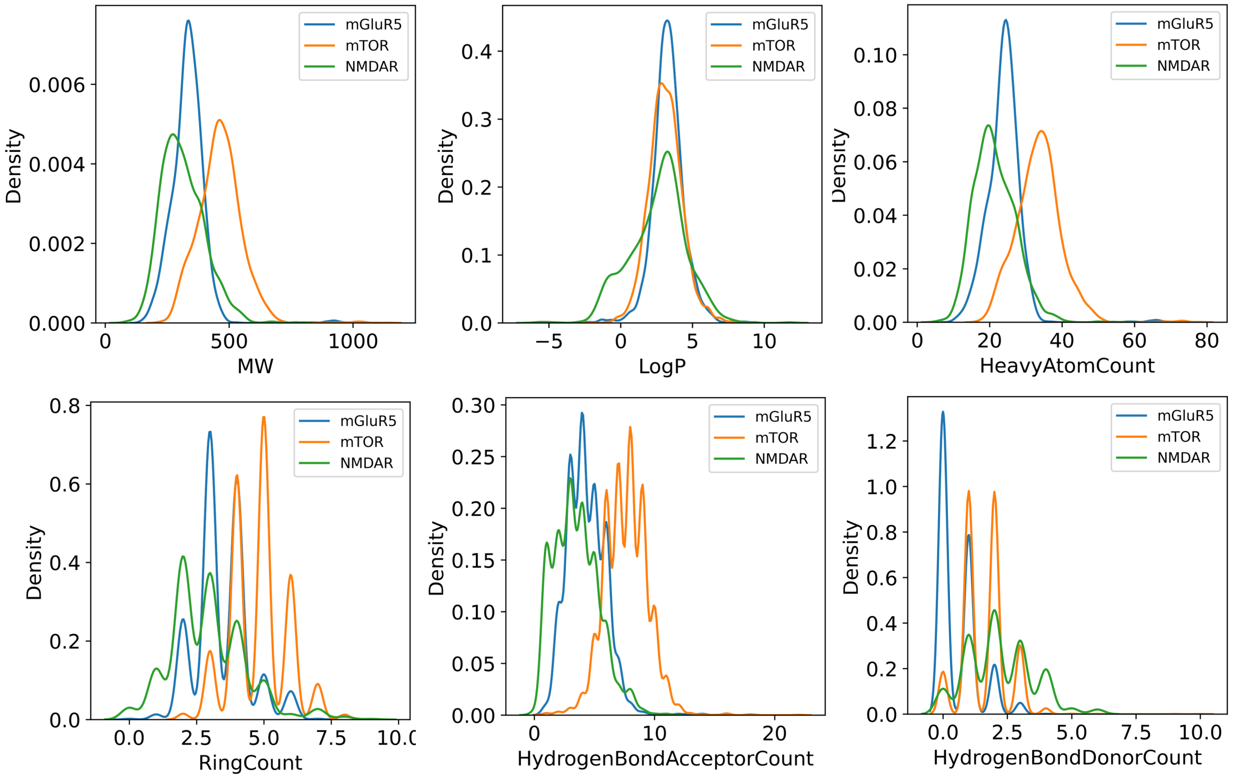

Figure S2: Distribution of Six Molecular Properties in the Training Set.

We constructed predictive models for binding affinity using inhibitor datasets obtained from the ChEMBL database. We then utilize these models to predict the binding affinity of drugs in DrugBank to these three targets. The compounds in these datasets are represented by SMILES strings, and their binding affinities (kcal/mol) serve as labels. The distribution of labels for each dataset is illustrated in Figure S1.

Appendix G Evaluation metrics

To assess the performance of our regression models, we employed two key metrics: the Pearson Correlation Coefficient (PCC) and the Root Mean Squared Error (RMSE). The definitions and formulas for these metrics are as follows: The Pearson Correlation Coefficient is defined for two vectors $x = (x_1, x_2, \ldots, x_n)$ and $y = (y_1, y_2, \ldots, y_n)$ as:

$$R = \text{PCC}(x, y) = \frac{\sum(x_i - \overline{x})(y_i - \overline{y})}{\sqrt{\sum(x_i - \overline{x})^2 \sum(y_i - \overline{y})^2}} \tag{17}$$

where $\overline{x}$ and $\overline{y}$ represent the mean of vectors x and y, respectively. The Root Mean Squared Error (RMSE) is calculated as:

$$\text{RMSE} = \sqrt{\frac{1}{n}\sum_{i=1}^{n}(y_i - \hat{y}_i)^2}, \tag{18}$$

where y_i is the true value and $\hat{y}_i$ is the predicted value for the i-th sample.

Appendix H Performance comparison of machine learning models

Table S4: Performance Comparison of Single Models and Consensus Models. This table provides a comprehensive comparison between the performance of individual single models and the combined consensus models, as measured by the Pearson Correlation Coefficient and Root Mean Square Error (RMSE). The models are differentiated based on the type of features used: Extended-Connectivity Fingerprints (ECFP), Autoencoder (AE), and Transformer-based (TF) features. Additionally, the consensus models are denoted by a plus symbol and represent an aggregation of predictions from various single models.

Model	mTOR	mGluR5	NMDAR
ECFP	0.852 (0.812)	0.734 (0.904)	0.773 (1.043)
AE	0.828 (0.881)	0.692 (0.966)	0.770 (1.058)
TF	0.835 (0.862)	0.704 (0.950)	0.776 (1.045)
TF+ECFP+AE	0.861 (0.801)	0.741 (0.910)	0.797 (1.014)
TF+ECFP	**0.876 (0.758)**	**0.754 (0.882)**	**0.799 (0.991)**

References

[1] Xiaqing Ma, Wenjie Du, Wenying Wang, Limin Luo, Min Huang, Haiyan Wang, Raozhou Lin, Zhongping Li, Haibo Shi, Tifei Yuan, et al. Persistent rheb-induced mtorc1 activation in spinal cord neurons induces hypersensitivity in neuropathic pain. *Cell death & disease*, 11(9):747, 2020.

[2] Haijuan Yang, Xiaolu Jiang, Buren Li, Hyo J Yang, Meredith Miller, Angela Yang, Ankita Dhar, and Nikola P Pavletich. Mechanisms of mtorc1 activation by rheb and inhibition by pras40. *Nature*, 552(7685):368–373, 2017.

[3] Ji-Tian Xu, Jian-Yuan Zhao, Xiuli Zhao, Davinna Ligons, Vinod Tiwari, Fidelis E Atianjoh, Chun-Yi Lee, Lingli Liang, Weidong Zang, Dolores Njoku, et al. Opioid receptor–triggered spinal mtorc1 activation contributes to morphine tolerance and hyperalgesia. *The Journal of clinical investigation*, 124(2):592–603, 2014.

[4] Jeffrey Bailey, Dzwokai Ma, and Karen K Szumlinski. Rapamycin attenuates the expression of cocaine-induced place preference and behavioral sensitization. *Addiction biology*, 17(2):248–258, 2012.

[5] Jinfang Wu, Sarah E McCallum, Stanley D Glick, and Yunfei Huang. Inhibition of the mammalian target of rapamycin pathway by rapamycin blocks cocaine-induced locomotor sensitization. *Neuroscience*, 172:104–109, 2011.

[6] Salman Zubedat and Irit Akirav. The involvement of cannabinoids and mtor in the reconsolidation of an emotional memory in the hippocampal–amygdala–insular circuit. *European neuropsychopharmacology*, 27(4):336–349, 2017.

[7] Jérémie Neasta, Sami Ben Hamida, Quinn Yowell, Sebastien Carnicella, and Dorit Ron. Role for mammalian target of rapamycin complex 1 signaling in neuroadaptations underlying alcohol-related disorders. *Proceedings of the National Academy of Sciences*, 107(46):20093–20098, 2010.

[8] Jeremie Neasta, Segev Barak, Sami Ben Hamida, and Dorit Ron. mtor complex 1: a key player in neuroadaptations induced by drugs of abuse. *Journal of neurochemistry*, 130(2):172–184, 2014.

[9] Rainer Spanagel. Alcoholism: a systems approach from molecular physiology to addictive behavior. *Physiological reviews*, 89(2):649–705, 2009.

[10] Beth M Hacker, James E Tomlinson, Gary A Wayman, Razia Sultana, Guy Chan, Enrique Villacres, Christine Disteche, and Daniel R Storm. Cloning, chromosomal mapping, and regulatory properties of the human type 9 adenylyl cyclase (adcy9). *Genomics*, 50(1):97–104, 1998.

[11] Andrés Couve, Philip Thomas, Andrew R Calver, Warren D Hirst, Menelas N Pangalos, Frank S Walsh, Trevor G Smart, and Stephen J Moss. Cyclic amp–dependent protein kinase phosphorylation facilitates gabab receptor–effector coupling. *Nature neuroscience*, 5(5):415–424, 2002.

[12] Jennifer Danielsson, Sarah Zaidi, Benjamin Kim, Hiromi Funayama, Peter D Yim, Dingbang Xu, Tilla S Worgall, George Gallos, and Charles W Emala. Airway epithelial cell release of gaba is regulated by protein kinase a. *Lung*, 194:401–408, 2016.

[13] M Katherine Kelm, Hugh E Criswell, and George R Breese. The role of protein kinase a in the ethanol-induced increase in spontaneous gaba release onto cerebellar purkinje neurons. *Journal of neurophysiology*, 100(6):3417–3428, 2008.

[14] Bernard J McDonald, Alessandra Amato, Christopher N Connolly, Dietmar Benke, Stephen J Moss, and Trevor G Smart. Adjacent phosphorylation sites on gabaa receptor β subunits determine regulation by camp-dependent protein kinase. *Nature neuroscience*, 1(1):23–28, 1998.

[15] Kushal Kumar, Sorabh Sharma, Puneet Kumar, and Rahul Deshmukh. Therapeutic potential of gabab receptor ligands in drug addiction, anxiety, depression and other cns disorders. *Pharmacology Biochemistry and Behavior*, 110:174–184, 2013.

[16] Sheila MS Sears and Sandra J Hewett. Influence of glutamate and gaba transport on brain excitatory/inhibitory balance. *Experimental Biology and Medicine*, 246(9):1069–1083, 2021.

[17] Zachary D Brodnik, Aashita Batra, Erik B Oleson, and Rodrigo A Espana. Local gabaa receptor-mediated suppression of dopamine release within the nucleus accumbens. *ACS chemical neuroscience*, 10(4):1978–1985, 2018.

[18] Bradley M Roberts, Natalie M Doig, Katherine R Brimblecombe, Emanuel F Lopes, Ruth E Siddorn, Sarah Threlfell, Natalie Connor-Robson, Nora Bengoa-Vergniory, Nicholas Pasternack, Richard Wade-Martins, et al. Gaba uptake transporters support dopamine release in dorsal striatum with maladaptive downregulation in a parkinsonism model. *Nature communications*, 11(1):4958, 2020.

[19] Mingzheng Wu, Zehua Li, Lei Liang, Pingchuan Ma, Dong Cui, Peng Chen, Genhao Wu, and Xue-Jun Song. Wnt signaling contributes to withdrawal symptoms from opioid receptor activation induced by morphine exposure or chronic inflammation. *Pain*, 161(3):532, 2020.

[20] Xin Liu, Chilman Bae, Bolong Liu, Yong-Mei Zhang, Xiangfu Zhou, Donghang Zhang, Cheng Zhou, Adriana DiBua, Livia Schutz, Martin Kaczocha, et al. Development of opioid-induced hyperalgesia depends on reactive astrocytes controlled by wnt5a signaling. *Molecular Psychiatry*, 28(2):767–779, 2023.

[21] Jeroen M Bugter, Nicola Fenderico, and Madelon M Maurice. Mutations and mechanisms of wnt pathway tumour suppressors in cancer. *Nature Reviews Cancer*, 21(1):5–21, 2021.

[22] Jiaqi Liu, Qing Xiao, Jiani Xiao, Chenxi Niu, Yuanyuan Li, Xiaojun Zhang, Zhengwei Zhou, Guang Shu, and Gang Yin. Wnt/β-catenin signalling: function, biological mechanisms, and therapeutic opportunities. *Signal transduction and targeted therapy*, 7(1):3, 2022.

[23] Akira Nishi, Shusuke Numata, Atsushi Tajima, Xiaolei Zhu, Koki Ito, Atsushi Saito, Yusuke Kato, Makoto Kinoshita, Shinji Shimodera, Shinji Ono, et al. De novo non-synonymous tbl1xr1 mutation alters wnt signaling activity. *Scientific reports*, 7(1):2887, 2017.

[24] Hylan C Moises, Konstantin I Rusin, and RL Macdonald. Mu-and kappa-opioid receptors selectively reduce the same transient components of high-threshold calcium current in rat dorsal root ganglion sensory neurons. *Journal of Neuroscience*, 14(10):5903–5916, 1994.

[25] Gerald W Zamponi and Terrance P Snutch. Modulating modulation: crosstalk between regulatory pathways of presynaptic calcium channels. *Molecular interventions*, 2(8):476, 2002.

[26] C König, O Gavrilova-Ruch, G Segond Von Banchet, R Bauer, M Grün, E Hirsch, I Rubio, S Schulz, SH Heinemann, HG Schaible, et al. Modulation of μ-opioid receptor desensitization in peripheral sensory neurons by phosphoinositide 3-kinase γ. *Neuroscience*, 169(1):449–454, 2010.

[27] Sreedhar Madishetti, Nadine Schneble, Christian König, Emilio Hirsch, Stefan Schulz, Jörg P Müller, and Reinhard Wetzker. Pi3kγ integrates c amp and akt signalling of the μ-opioid receptor. *British journal of pharmacology*, 171(13):3328–3337, 2014.

[28] Mayumi Miyatake, Tal J Rubinstein, Gregory P McLennan, Mariana M Belcheva, and Carmine J Coscia. Inhibition of egf-induced erk/map kinase-mediated astrocyte proliferation by μ opioids: integration of g protein and β-arrestin 2-dependent pathways. *Journal of neurochemistry*, 110(2):662–674, 2009.

[29] Nahid A Shahabi, Kathy McAllen, and Burt M Sharp. δ opioid receptors stimulate akt-dependent phosphorylation of c-jun in t cells. *Journal of Pharmacology and Experimental Therapeutics*, 316(2):933–939, 2006.

[30] Miao Tan, Wendy M Walwyn, Christopher J Evans, and Cui-Wei Xie. p38 mapk and β-arrestin 2 mediate functional interactions between endogenous μ-opioid and α2a-adrenergic receptors in neurons. *Journal of biological chemistry*, 284(10):6270–6281, 2009.

[31] Noboru Hiroi, Jennifer R Brown, Colin N Haile, Hong Ye, Michael E Greenberg, and Eric J Nestler. Fos b mutant mice: loss of chronic cocaine induction of fos-related proteins and heightened sensitivity to cocaine's psychomotor and rewarding effects. *Proceedings of the National Academy of Sciences*, 94(19):10397–10402, 1997.

[32] Jianhua Zhang, Lu Zhang, Hongyuan Jiao, Qi Zhang, Dongsheng Zhang, Danwen Lou, Jonathan L Katz, and Ming Xu. c-fos facilitates the acquisition and extinction of cocaine-induced persistent changes. *Journal of Neuroscience*, 26(51):13287–13296, 2006.

[33] Colleen A McClung and Eric J Nestler. Regulation of gene expression and cocaine reward by creb and δfosb. *Nature neuroscience*, 6(11):1208–1215, 2003.

[34] BA Nic Dhonnchadha, BF Lovascio, N Shrestha, A Lin, KA Leite-Morris, HY Man, GB Kaplan, and KM Kantak. Changes in expression of c-fos protein following cocaine-cue extinction learning. *Behavioural brain research*, 234(1):100–106, 2012.

[35] Ming Xu. c-fos is an intracellular regulator of cocaine-induced long-term changes. *Annals of the New York Academy of Sciences*, 1139(1):1–9, 2008.

[36] Huynh Nhu Mai, Yoon Hee Chung, Eun-Joo Shin, Naveen Sharma, Ji Hoon Jeong, Choon-Gon Jang, Kuniaki Saito, Toshitaka Nabeshima, Dora Reglodi, and Hyoung-Chun Kim. Il-6 knockout mice are protected from cocaine-induced kindling behaviors; possible involvement of jak2/stat3 and pacap signalings. *Food and Chemical Toxicology*, 116:249–263, 2018.

[37] Fernanda Pedrotti Moreira, João Ricardo Carvalho Medeiros, Alfredo Cardoso Lhullier, Luciano Dias de Mattos Souza, Karen Jansen, Luis Valmor Portela, Diogo R Lara, Ricardo Azevedo da Silva, Carolina David Wiener, and Jean Pierre Oses. Cocaine abuse and effects in the serum levels of cytokines il-6 and il-10. *Drug and alcohol dependence*, 158:181–185, 2016.

[38] Bruna S da Silva, Renata B Cupertino, Jaqueline B Schuch, Djenifer B Kappel, Breno Sanvicente-Vieira, Cibele E Bandeira, Lisia von Diemen, Felix HP Kessler, Eugenio H Grevet, Rodrigo Grassi-Oliveira, et al. The association between syt1-rs2251214 and cocaine use disorder further supports its role in psychiatry. *Progress in Neuro-Psychopharmacology and Biological Psychiatry*, 94:109642, 2019.

[39] Thiago Wendt Viola, Jaqueline Bohrer Schuch, Diego Luiz Rovaris, Rafael Genovese, Lucca Tondo, Breno Sanvicente-Vieira, Aline Zaparte, Renata Basso Cupertino, Bruna Santos da Silva, Claiton Hen-

rique Dotto Bau, et al. Association between cognitive performance and syt1-rs2251214 among women with cocaine use disorder. *Journal of Neural Transmission*, 126:1707–1711, 2019.

[40] Anita Sidhu, Christophe Wersinger, CHARBEL E-H MOUSSA, and Philippe Vernier. The role of α-synuclein in both neuroprotection and neurodegeneration. *Annals of the New York Academy of Sciences*, 1035(1):250–270, 2004.

[41] Yujing Qin, Qinjie Ouyang, John Pablo, and Deborah C Mash. Cocaine abuse elevates alpha-synuclein and dopamine transporter levels in the human striatum. *Neuroreport*, 16(13):1489–1493, 2005.

[42] Tatiana Foroud, Leah Flury Wetherill, Tiebing Liang, Danielle M Dick, Victor Hesselbrock, John Kramer, John Nurnberger, Marc Schuckit, Lucinda Carr, Bernice Porjesz, et al. Association of alcohol craving with α-synuclein (snca). *Alcoholism: Clinical and Experimental Research*, 31(4):537–545, 2007.

[43] Alexey Bingor, Matityahu Azriel, Lavi Amiad, and Rami Yaka. Potentiated response of erk/mapk signaling is associated with prolonged withdrawal from cocaine behavioral sensitization. *Journal of Molecular Neuroscience*, pages 1–8, 2021.

[44] Wei-Lun Sun, Pamela M Quizon, and Jun Zhu. Molecular mechanism: Erk signaling, drug addiction, and behavioral effects. *Progress in molecular biology and translational science*, 137:1–40, 2016.

[45] Keren Bachi, Venkatesh Mani, Devi Jeyachandran, Zahi A Fayad, Rita Z Goldstein, and Nelly Alia-Klein. Vascular disease in cocaine addiction. *Atherosclerosis*, 262:154–162, 2017.

[46] Bryan G Schwartz, Shereif Rezkalla, and Robert A Kloner. Cardiovascular effects of cocaine. *Circulation*, 122(24):2558–2569, 2010.

[47] Gil M Lewitus, Sarah C Konefal, Andrew D Greenhalgh, Horia Pribiag, Keanan Augereau, and David Stellwagen. Microglial tnf-α suppresses cocaine-induced plasticity and behavioral sensitization. *Neuron*, 90(3):483–491, 2016.

[48] Chia-Hsiang Chen, Chia-Chun Huang, and Ding-Lieh Liao. Association analysis of gabrb3 promoter variants with heroin dependence. *PLoS One*, 9(7):e102227, 2014.

[49] Ernest P Noble, Xuxian Zhang, Terry Ritchie, Bruce R Lawford, Stella C Grosser, Ross McD Young, and Robert S Sparkes. D2 dopamine receptor and gabaa receptor β3 subunit genes and alcoholism. *Psychiatry Research*, 81(2):133–147, 1998.

[50] Jiuzhou Song, Daniel L Koller, Tatiana Foroud, Kristie Carr, Jinghua Zhao, John Rice, John I Nurnberger Jr, Henri Begleiter, Bernice Porjesz, Tom L Smith, et al. Association of gabaa receptors and alcohol dependence and the effects of genetic imprinting. *American Journal of Medical Genetics Part B: Neuropsychiatric Genetics*, 117(1):39–45, 2003.

[51] Ross McD Young, Bruce R Lawford, Gerald FX Feeney, Terry Ritchie, and Ernest P Noble. Alcohol-related expectancies are associated with the d2 dopamine receptor and gabaa receptor β3 subunit genes. *Psychiatry research*, 127(3):171–183, 2004.

[52] Arshad H Khan, Jared R Bagley, Nathan LaPierre, Carlos Gonzalez-Figueroa, Tadeo C Spencer, Mudra Choudhury, Xinshu Xiao, Eleazar Eskin, James D Jentsch, and Desmond J Smith. Genetic pathways regulating the longitudinal acquisition of cocaine self-administration in a panel of inbred and recombinant inbred mice. *Cell reports*, 42(8), 2023.

[53] Bao-Zhu Yang, Shizhong Han, Henry R Kranzler, Lindsay A Farrer, and Joel Gelernter. A genomewide linkage scan of cocaine dependence and major depressive episode in two populations. *Neuropsychopharmacology*, 36(12):2422–2430, 2011.

[54] Annalisa Buniello, Jacqueline A L MacArthur, Maria Cerezo, Laura W Harris, James Hayhurst, Cinzia Malangone, Aoife McMahon, Joannella Morales, Edward Mountjoy, Elliot Sollis, et al. The nhgri-ebi gwas catalog of published genome-wide association studies, targeted arrays and summary statistics 2019. *Nucleic acids research*, 47(D1):D1005–D1012, 2019.

[55] Takuya Nishimura, Atsutaka Kubosaki, Yoichiro Ito, and Abner L Notkins. Disturbances in the secretion of neurotransmitters in ia-2/ia-2β null mice: changes in behavior, learning and lifespan. *Neuroscience*, 159(2):427–437, 2009.

[56] L David Porter, Hend Ibrahim, Lynn Taylor, and Norman P Curthoys. Complexity and species variation of the kidney-type glutaminase gene. *Physiological genomics*, 9(3):157–166, 2002.

[57] Javier Márquez, José A Campos-Sandoval, Ana Peñalver, José M Matés, Juan A Segura, Eduardo Blanco, Francisco J Alonso, and Fernando Rodríguez de Fonseca. Glutamate and brain glutaminases in drug addiction. *Neurochemical research*, 42:846–857, 2017.

[58] Manoranjan S D'Souza. Glutamatergic transmission in drug reward: implications for drug addiction. *Frontiers in neuroscience*, 9:404, 2015.

[59] Michail Vikelis and Dimos D Mitsikostas. The role of glutamate and its receptors in migraine. *CNS & Neurological Disorders-Drug Targets (Formerly Current Drug Targets-CNS & Neurological Disorders)*, 6(4):251–257, 2007.

[60] Camron D Bryant, Shoshana Eitan, Kevin Sinchak, Michael S Fanselow, and Christopher J Evans. Nmda receptor antagonism disrupts the development of morphine analgesic tolerance in male, but not female c57bl/6j mice. *American Journal of Physiology-Regulatory, Integrative and Comparative Physiology*, 291(2):R315–R326, 2006.

[61] Claire M Corbett, Emily ND Miller, and Jessica A Loweth. mglu5 inhibition in the basolateral amygdala prevents estrous cycle-dependent changes in cue-induced cocaine seeking. *Addiction neuroscience*, 5:100055, 2023.

[62] Michael J Glass. Opioid dependence and nmda receptors. *ILAR journal*, 52(3):342–351, 2011.

[63] Hideaki Kato, Minoru Narita, Kan Miyoshi, Megumi Asato, Nana Hareyama, Hiroyuki Nozaki, Tomoe Takagi, Masami Suzuki, and T Suzuki. Implication of src family kinase-dependent phosphorylation of nr2b subunit-containing nmda receptor in the rewarding effect of morphine. *Nihon shinkei seishin yakurigaku zasshi= Japanese journal of psychopharmacology*, 26(3):119–124, 2006.

[64] Hideaki Kato, Minoru Narita, Masami Suzuki, Kanji Yoshimoto, Masahiro Yasuhara, and Tsutomu Suzuki. Role of tyrosine kinase-dependent phosphorylation of nr2b subunit-containing nmda receptor in morphine reward. *Nihon Arukoru Yakubutsu Igakkai Zasshi= Japanese Journal of Alcohol Studies & Drug Dependence*, 42(1):13–20, 2007.

[65] Yoan Mihov and Gregor Hasler. Negative allosteric modulators of metabotropic glutamate receptors subtype 5 in addiction: a therapeutic window. *International Journal of Neuropsychopharmacology*, 19(7):pyw002, 2016.

[66] Michael C Salling, Alexander Grassetti, Vincent P Ferrera, Diana Martinez, and Richard W Foltin. Negative allosteric modulation of metabotropic glutamate receptor 5 attenuates alcohol self-administration in baboons. *Pharmacology Biochemistry and Behavior*, 208:173227, 2021.

[67] Cassia N Cearley, Kelly Blindheim, Barbara A Sorg, James M Krueger, and Lynn Churchill. Acute cocaine increases interleukin-1β mrna and immunoreactive cells in the cortex and nucleus accumbens. *Neurochemical research*, 36:686–692, 2011.

[68] Jorge Montesinos, Estela Castilla-Ortega, Laura Sánchez-Marín, Sandra Montagud-Romero, Pedro Araos, María Pedraz, Óscar Porras-Perales, Nuria García-Marchena, Antonia Serrano, Juan Suárez, et al. Cocaine-induced changes in cx3cl1 and inflammatory signaling pathways in the hippocampus: Association with il1β. *Neuropharmacology*, 162:107840, 2020.

[69] Liang Liu, Mark R Hutchinson, Jason M White, Andrew A Somogyi, and Janet K Coller. Association of il-1b genetic polymorphisms with an increased risk of opioid and alcohol dependence. *Pharmacogenetics and genomics*, 19(11):869–876, 2009.

[70] Guoli Xiong, Zhenxing Wu, Jiacai Yi, Li Fu, Zhijiang Yang, Changyu Hsieh, Mingzhu Yin, Xiangxiang Zeng, Chengkun Wu, Aiping Lu, et al. Admetlab 2.0: an integrated online platform for accurate and comprehensive predictions of admet properties. *Nucleic Acids Research*, 49(W1):W5–W14, 2021.

[71] Afra Zomorodian and Gunnar Carlsson. Computing persistent homology. In *Proceedings of the twentieth annual symposium on Computational geometry*, pages 347–356, 2004.

[72] Dong Chen, Jian Liu, Jie Wu, Guo-Wei Wei, Feng Pan, and Shing-Tung Yau. Path topology in molecular and materials sciences. *The Journal of Physical Chemistry Letters*, 14(4):954–964, 2023.

[73] David Mendez, Anna Gaulton, A Patrícia Bento, Jon Chambers, Marleen De Veij, Eloy Félix, María Paula Magariños, Juan F Mosquera, Prudence Mutowo, Michał Nowotka, et al. Chembl: towards direct deposition of bioassay data. *Nucleic acids research*, 47(D1):D930–D940, 2019.

[74] Tuomo Kalliokoski, Christian Kramer, Anna Vulpetti, and Peter Gedeck. Comparability of mixed ic50 data–a statistical analysis. *PloS one*, 8(4):e61007, 2013.

[75] David S Wishart, Yannick D Feunang, An C Guo, Elvis J Lo, Ana Marcu, Jason R Grant, Tanvir Sajed, Daniel Johnson, Carin Li, Zinat Sayeeda, et al. Drugbank 5.0: a major update to the drugbank database for 2018. *Nucleic acids research*, 46(D1):D1074–D1082, 2018.

[76] Dong Chen, Jiaxin Zheng, Guo-Wei Wei, and Feng Pan. Extracting predictive representations from hundreds of millions of molecules. *The journal of physical chemistry letters*, 12(44):10793–10801, 2021.

[77] Robin Winter, Floriane Montanari, Frank Noé, and Djork-Arné Clevert. Learning continuous and data-driven molecular descriptors by translating equivalent chemical representations. *Chemical science*, 10(6):1692–1701, 2019.

[78] David Rogers and Mathew Hahn. Extended-connectivity fingerprints. *Journal of chemical information and modeling*, 50(5):742–754, 2010.

[79] Sunghwan Kim, Paul A Thiessen, Evan E Bolton, Jie Chen, Gang Fu, Asta Gindulyte, Lianyi Han, Jane He, Siqian He, Benjamin A Shoemaker, et al. Pubchem substance and compound databases. *Nucleic acids research*, 44(D1):D1202–D1213, 2016.

[80] John J Irwin and Brian K Shoichet. Zinc- a free database of commercially available compounds for virtual screening. *Journal of chemical information and modeling*, 45(1):177–182, 2005.

[81] Jerome H Friedman. Greedy function approximation: a gradient boosting machine. *Annals of statistics*, pages 1189–1232, 2001.

[82] Guolin Ke, Qi Meng, Thomas Finley, Taifeng Wang, Wei Chen, Weidong Ma, Qiwei Ye, and Tie-Yan Liu. Lightgbm: A highly efficient gradient boosting decision tree. *Advances in neural information processing systems*, 30, 2017.
